# Statistical parity-time-symmetric lasing in an optical fibre network

**DOI:** 10.1038/s41467-017-00958-x

**Published:** 2017-11-07

**Authors:** Ali K. Jahromi, Absar U. Hassan, Demetrios N. Christodoulides, Ayman F. Abouraddy

**Affiliations:** CREOL, The College of Optics and Photonics, University of Central Florida, Orlando, FL 32816 USA

## Abstract

Parity-time (PT)-symmetry in optics is a condition whereby the real and imaginary parts of the refractive index across a photonic structure are deliberately balanced. This balance can lead to interesting optical phenomena, such as unidirectional invisibility, loss-induced lasing, single-mode lasing from multimode resonators, and non-reciprocal effects in conjunction with nonlinearities. Because PT-symmetry has been thought of as fragile, experimental realisations to date have been usually restricted to on-chip micro-devices. Here, we demonstrate that certain features of PT-symmetry are sufficiently robust to survive the statistical fluctuations associated with a macroscopic optical cavity. We examine the lasing dynamics in optical fibre-based coupled cavities more than a kilometre in length with balanced gain and loss. Although fluctuations can detune the cavity by more than the free spectral range, the behaviour of the lasing threshold and the laser power is that expected from a PT-stable system. Furthermore, we observe a statistical symmetry breaking upon varying the cavity loss.

## Abstract (web summary)

Parity-time-symmetric optical systems have so far only been realized using microscopic cavities because the necessary gain-loss balance is thought to be fragile with regard to statistical fluctuations. Here, Jahromi et al.. demonstrate PT-symmetric lasing using kilometre-long fibre cavities.

## Introduction

Since their mathematical inception^1, 2^, non-Hermitian parity-time (PT) -symmetric notions have found manifestations in many diverse physical embodiments, ranging from photonics^3–9^ to acoustics^10, 11^, phononics^12^ and even electronics^13, 14^. Nevertheless, optics has proven to date to be the most convenient platform for the realisation of PT-symmetry. In large part, the suitability of optics is a consequence of the facile deviation from Hermiticity achieved by adding optical loss (attenuation) or gain (amplification) to an initially unitary (lossless) system. These investigations have led to the observation of a number of counter-intuitive effects such as loss-induced transparency^15^, lasing suppression with increased gain^16^ and the revival of lasing with increased loss^17^. Furthermore, phenomena such as double refraction, power oscillations and solitons have been observed in photonic lattices^18, 19^. Recently perfect absorption has been suggested^20^ and realized^21^ in PT-symmetric configurations in addition to a burgeoning effort on PT-metasurfaces^22–27^. Furthermore, PT-symmetry has been employed to achieve one-sided invisibility^28, 29^, demonstrate unidirectional scattering and absorption^30, 31^, construct mode-selective lasers^32, 33^ and realise on-chip unidirectional devices^34, 35^. Moreover, ramifications of these concepts are currently being extended to the emerging field of topological photonics^36–38^ and ultra-sensitive measurement devices^39–41^.

Exact PT-symmetry is achieved by arranging a delicate balance deliberately introduced into the spatial distribution of the complex refractive index throughout the system. In general, it is required to simultaneously maintain the real part of the refractive index symmetric while the imaginary part (corresponding to gain or loss) is kept anti-symmetric under inversion^3, 4^. Note however, that PT-symmetry appears to be only a sufficient condition for the effects described above. Indeed, it has been shown that any patterned gain-loss structure in a deterministic scheme may exhibit counter-intuitive PT-symmetry-related phenomena^42^. Because establishing a PT-symmetric refractive index distribution imposes stringent fabrication requirements, experimental realisations to date have been usually restricted to on-chip micro-devices. To date, optical demonstrations of PT-symmetry have focused on micro-structures realized on a chip ranging from coupled semiconductor^15^ or photorefractive waveguides^5^ to coupled ring resonators in InP^32, 43^, quantum cascade disk-lasers^16^ or erbium-doped silica^34, 35^. The large free spectral range (FSR) associated with micro-devices helps isolate a single or a few relevant resonant modes in a compact and stable manner, thereby justifying a fully deterministic theoretical treatment. However, in larger optical systems such as fibre networks, the FSR can be so small that unavoidable fluctuations lead to detuning of the resonances between sub-systems – potentially reaching a full FSR. In view of the above, it is an open question whether signatures of PT-symmetry are retained in such large-scale settings.

Here we demonstrate that many features of PT-symmetry are sufficiently robust so as to survive the statistical fluctuations associated with macroscopic fibre cavities – even ones having a length in excess of 1 km. Starting from a generic linear PT-symmetric laser cavity model, we construct a conceptually analogous lumped-component model that we experimentally realise in a single-mode-fibre cavity. Coherent coupling and feedback from the interfaces in the traditional model are replaced by partially reflective fibre Bragg mirrors connecting two sub-cavities, in which optical amplification and attenuation are provided by localised components in lieu of the distributed gain and loss used in previous approaches. In such an arrangement, the gain-loss balance is readily maintained and varied deterministically, but the sub-cavity phases cannot be held fixed due to unavoidable fluctuations in such a large system – thereby leading to resonance detuning. To study non-Hermitian phenomena in such large-scale settings, we first theoretically analyse an ideal zero-detuned cavity geometry using a mean-field saturable model to obtain two distinct steady-state nonlinear supermodes. We then find that even in the presence of a random detuning, the system behaviour still mimics this zero-detuned ideal scenario. In this case, although the supermodes are not formally equivalent to those when the detuning is absent, the existence of unbroken and broken PT-symmetry phases can still be inferred. In addition, a gradual phase transition between these phases can also take place despite random fluctuations in the cavity resonances. We demonstrate experimentally and theoretically that the lasing threshold and the post-lasing output-power scaling in the PT-symmetric configuration survives the statistical detuning effects of the sub-cavity resonances – even when this detuning spans the full FSR. We present the first quantitative identification of lasing thresholds and broken and unbroken PT-symmetric lasing phases, which is made possible by the unambiguous separation of the power emitted by the gain and loss sub-cavities. Furthermore, we find that although detuning precludes the existence of an exact unbroken PT-symmetric phase, observation of the signature of symmetry breaking is nevertheless enabled through tuning the attenuation of the loss sub-cavity. As the loss is monotonically increased, a transition occurs from a phase in which the lasing power emitted from the gain side decays to one in which the lasing power increases with increased loss. We attribute this aspect to the presence of an exceptional point in this non-Hermitian arrangement. Our results thus extend the manifestation of non-Hermitian effects in large-scale non-deterministic settings. The demonstrated robustness of PT-symmetry in macroscopic fibre systems could pave the way to applications of such concepts in telecommunications and fibre lasers.

## Results

### Lumped-component model of a photonic PT-symmetric system

**Fig. 1:**
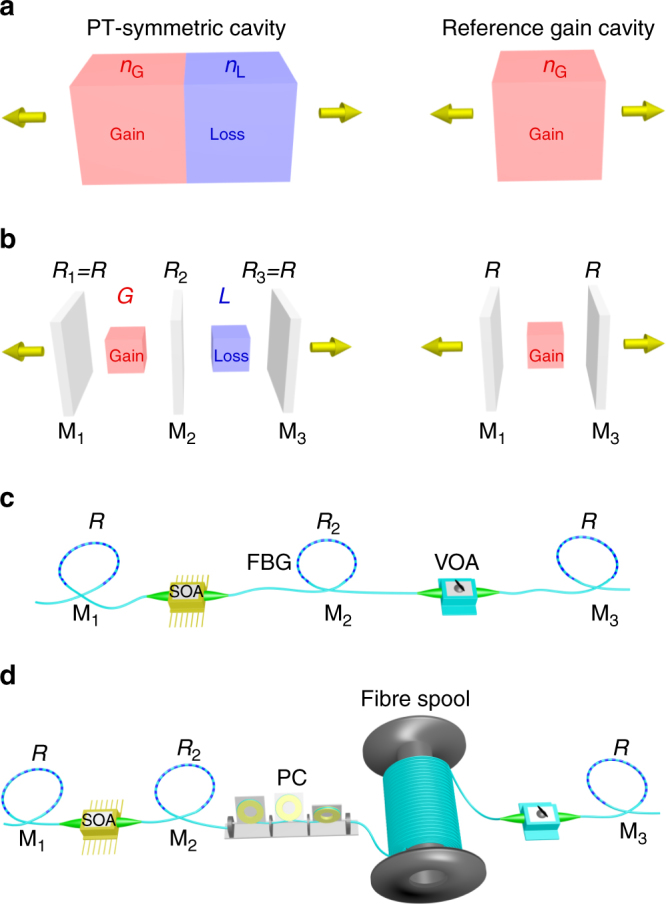
A lumped-component model of a PT-symmetric laser cavity. **a**, A PT-symmetric structure formed of two homogeneous layers of refractive indices *n*_g_ and *n*_*l*_, (corresponding to optical gain and loss, respectively) and equal thicknesses. PT-symmetry requires ${n_{\rm{g}}} = n_\ell ^*$. As a reference, the gain layer alone (corresponding to the reference cavity in Table 1) after removing the loss layer is shown on the right. **b**, A discrete model composed of lumped components to replace the continuum model in **a**: the interfaces are replaced with localised mirrors M_1_, M_2_ and M_3_, with reflectivities *R*_1_, *R*_2_ and *R*_3_, respectively. The distributed gain and loss are replaced with an amplifier (amplification factor *G*) and an attenuator (attenuation factor ${\cal L}$), respectively. PT-symmetry requires that *R*_1_=*R*_3_=*R* and $G{\cal L} = 1$. The cavity corresponding to the gain layer alone is formed of the side mirrors containing the amplifier. **c**, Schematic representation of an experimental realisation of the PT-symmetric structured cavity shown on the left in **b** using single-mode optical fibres. Specially designed FBGs are used as partially reflecting mirrors with reflectivies *R*, *R*_2_, and *R* from left to right. Gain is provided by a SOA and attenuation by a VOA. **d**, Optical setup in **c** after inserting an additional 1-km-long fibre spool. A polarisation controller (PC) is added to maintain the state of polarisation throughout the cavity

We start by abstracting from an archetypical optical PT-symmetric configuration (Fig. 1a), an equivalent discrete lumped-component system (Fig. 1b-d). The arrangement shown in Fig. 1a consists of equal lengths of homogeneous materials of refractive indices *n*_G_ and *n*_L_ in intimate contact and surrounded with symmetric external media. The imaginary part of the index corresponds to either optical loss (*n*_L_) or gain (*n*_G_), depending on its sign. If the indices satisfy $n_{\rm{G}}^* = {n_{\rm{L}}}$, then the structure is said to be PT-symmetric. This condition entails that the real part of the refractive index has an even distribution (it is equal in both layers), whereas the imaginary part has an odd distribution (optical gain in one layer and matching losses in the other). Index discontinuities at all three interfaces provide reflection that is particularly weak at the interface between the two layers (where it depends on only the contrast between the imaginary components of *n*_G_ and *n*_L_) – resulting in strong coupling between the two layers. Despite the simplicity of this fundamental model, it has not been experimentally realized to date – in part due to the constraints placed by the Kramers–Kronig relations on the commensurate values of the real and imaginary components of the refractive index of any material^44^. To date, many physical realisations of PT-symmetric cavities have focused instead on other micro-systems such as coupled ring cavities or parallel waveguides.

The optical structure shown in Fig. 1b that comprises two coupled sub-cavities is conceptually equivalent to that in Fig. 1a. Fresnel reflection at the interfaces is replaced by partially reflecting mirrors: outer symmetric mirrors M_1_ and M_3_ having equal reflectivities *R*_1_=*R*_2_=*R* that correspond to the interfaces with the external media, and a middle mirror M_2_ of reflectivity *R*_2_ that couples the two sub-cavities and corresponds to the interface between the gain and loss layers. Discrete optical amplifiers and attenuators provide single-pass amplification *G* and attenuation ${\cal L}$ in the sub-cavities. Crucially, in such a configuration the reflections are no longer constrained by the physical limitations on the refractive indices of materials as dictated by the Kramers–Kronig relations. Instead, coherent feedback between the sub-cavities becomes independent of the gain/loss contrast.

In the lumped-component model, balanced gain and loss corresponds to $G{\cal L} = 1$, removing attenuation altogether from the lossy sub-cavity corresponds to ${\cal L} = 1$ (gain-lossless configuration in Table 1), and an infinite attenuation to ${\cal L} = 0$ (gain sub-cavity in Table 1). From this perspective, the PT-symmetric arrangement is a particular element in a continuum of possibilities, where ${\cal L}$ is varied and *G* is held fixed. This family of structured laser cavities can be viewed as a result of inserting passive elements (the mirror M_2_ and the attenuation ${\cal L}$) into a symmetric reference cavity consisting of an amplifying gain element *G* between two mirrors M_1_ and M_3_ having an equal reflectivity *R* (Fig. 1a, b; reference gain cavity) – corresponding to the addition of a loss layer to the gain layer. A question naturally arises whether the establishment of PT-symmetry by inserting a gain-balancing loss will inevitably raise the lasing threshold of this structured cavity with respect to that of a reference cavity where the loss is eliminated? We proceed to show that this is not always the case.

**Table 1:** Comparison of the lasing threshold of a PT-symmetric cavity with those for other configurations after removing the loss element

	

Three cavity models are considered here: a, a PT-symmetric cavity comprising sub-cavities with balanced gain and loss $G{\cal L} = 1$; b, the same cavity configuration in (a) after removing the loss component from its sub-cavity; c, a reference cavity with symmetric mirror reflectivities *R* containing only a gain element with amplification *G*. In all cases, we examine the measured and expected lasing threshold as *R*_2_ is varied. All the lasing threshold values in the table are in dB, the value *R*=82% is held fixed, and the average error in the measured thresholds is  ≈ 0.2 dB. The values of the threshold for the reference cavity (c) are independent of *R*_2_

### Experimental realisation in a PT-symmetric fibre-based cavity

To realise the lumped-component PT-symmetric structure shown in Fig. 1b, we have constructed a C-band single-mode-fibre-based cavity in which all the degrees of freedom are independently accessible, as illustrated in Fig. 1c. Gain is produced by a fibre-pigtailed semiconductor optical amplifier (SOA), the loss is induced by a variable optical attenuator (VOA), and optical feedback is provided by custom-made fibre Bragg gratings (FBGs) with desired reflectivity, central wavelength, and bandwidth (Methods). A single polarisation is maintained by utilising a polarisation-sensitive SOA and polarisation-maintaining optical components. Here we keep the reflectivities of the side mirrors fixed at *R* ≈ 82% (left and right external FBGs M_1_ and M_3_), and vary *R*_2_ from 7 to 99% for the intra-sub-cavity coupling FBG M_2_.

We first measure the lasing threshold of the PT-symmetric configuration by gradually increasing the contrast between *G* and ${\cal L}$, while maintaining the balanced condition $G{\cal L} = 1$ until lasing is initiated. The PT-symmetric lasing thresholds *G*=*G*_PT_ for different *R*_2_ are listed in Table 1. Measurements of the thresholds at the two limits of the above-described continuum of arrangements while varying ${\cal L}$ are also listed. At ${\cal L} = 1$ (gain-lossless), the threshold *G*_0_ is always less than *G*_PT_; whereas for ${\cal L} = 0$ (gain sub-cavity), the threshold *G*_open_ is always higher than *G*_PT_. The PT-symmetric cavity may have higher or lower threshold *G*_PT_ in comparison to the threshold *G*_Ref_ for the reference cavity (after removing the passive elements M_2_ and ${\cal L}$). Measurements of the thresholds in all four cavity configurations while varying *R*_2_ are compared to theoretical values obtained by identifying the poles of the cavity transmission (Supplementary Note 1),

1 $${G_{{\rm{PT}}}} = \frac{{1 - R}}{{2\tilde R}} + \sqrt {1 + {{\left( {\frac{{1 - R}}{{2\tilde R}}} \right)}^{\!\!\!2}}} ,\,{G_0} = \frac{{1 + \tilde R}}{{R + \tilde R}},\,{G_{{\rm{open}}}} = \frac{1}{{\tilde R}},\,{G_{{\rm{Ref}}}} = \frac{1}{R},$$

where $\tilde R = \sqrt {R{R_2}}$. These expressions remain unaffected whether the cavities are deterministic or if we assume randomly varying phases inserted in the sub-cavities.

We would like to stress the reason behind comparing *G*_PT_ with *G*_Ref_ and not the other three-mirror systems. Previously considered models have usually dealt with coupled-disk or coupled-cavity lasers that are evanescently coupled. In this scenario, once one sub-cavity is removed from the system (the lossy cavity, for example), the coupling also disappears. Comparing this to our linear cavity arrangement, the coupling is provided by the central mirror *R*_2_. Hence, in order to remove the loss sub-cavity, a fair comparison can only be made if the coupling mirror *R*_2_ is also removed, which is the reason for comparing *G*_PT_ with *G*_Ref_.

**Fig. 2:**
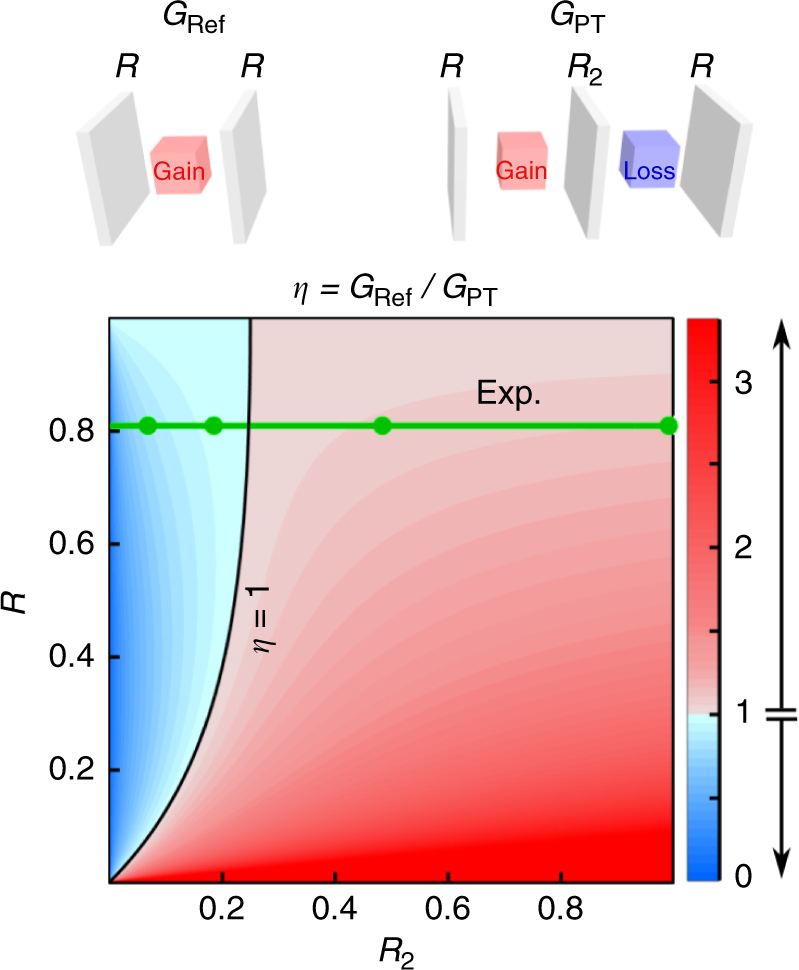
Comparison of the thresholds from a PT-symmetric cavity and a reference cavity. The threshold-reduction factor $\eta = {G_{{\rm{Ref}}}}/{G_{{\rm{PT}}}}$, where *G*_Ref_ and *G*_PT_ are the lasing thresholds for the reference gain-only cavity and the PT-symmetric cavity configurations, respectively, shown schematically at the top. We plot *η* with *R* and *R*_2_, and two colour palettes are used to distinguish the regime of PT-enhanced threshold where introducing the gain-balancing loss reduces the lasing threshold with respect to that of the reference gain-only cavity (*η*>1, red palette), and PT-diminished threshold (*η*<1, blue palette), delineated by a black curve (*η*=1). The horizontal green line corresponds to the experimental value *R*=0.82, and the circles to the experimental values of *R*_2_ (see Table 1)

The differences between the thresholds of the distinct cavity configurations listed in Table 1 are most prominent at low *R*_2_, whereupon the two sub-cavities are strongly coupled. As *R*_2_ increases, the differences between *G*_PT_, *G*_0_, and *G*_open_ are reduced monotonically and ultimately vanish as the amplifying sub-cavity is effectively isolated from its attenuating counterpart. In comparing *G*_PT_ to *G*_Ref_; however, we find two regimes while varying *R*_2_. At low *R*_2_ (strong coupling), we have *G*_PT_>*G*_Ref_, whereas increasing *R*_2_ can result in *G*_PT_<*G*_Ref_, therefore indicating that introducing gain-balancing loss into the reference cavity–counter-intuitively–may help reduce the lasing threshold. The advantage of a PT-symmetric cavity over the reference gain cavity is brought out in Fig. 2, where we plot the threshold-reduction factor *η*=*G*_Ref_/*G*_PT_. This ratio is unity when *R*_2_=*R*/(1+*R*)^2^, which is identified by the black curve in Fig. 2 that divides the parameter space into two regions: *η*>1 where PT-symmetry helps lower the lasing threshold, and *η*<1 where it does not. The lasing threshold is reduced despite introducing gain-balancing loss in the cavity whenever *R*_2_ and *R* are judiciously selected.

### Theoretical model for the coupled-cavity laser system

The expressions for lasing thresholds in Eq. (1) are obtained via the transfer matrix method that posits a linear model for all the optical components (Supplementary Note 1). As such, this approach is not suitable for describing the lasing dynamics whereupon the fields may experience exponential growth. In this nonlinear regime, we employ a mean-field temporal coupled-mode approach^45^ (Supplementary Note 2). The averaged field amplitudes *a* and *b* in the gain sub-cavity and the loss sub-cavity, respectively, are coupled through

2 $$\frac{{da}}{{dt}} = - {\gamma _1}a + i\frac{{\rm{\Delta }}}{2}a + \frac{g}{{1 + {{\left| a \right|}^2}}}a + i\kappa b,$$

3 $$\frac{{db}}{{dt}} = - {\gamma _2}b - i\frac{{\rm{\Delta }}}{2}b + i\kappa a.$$

**Fig. 3:**
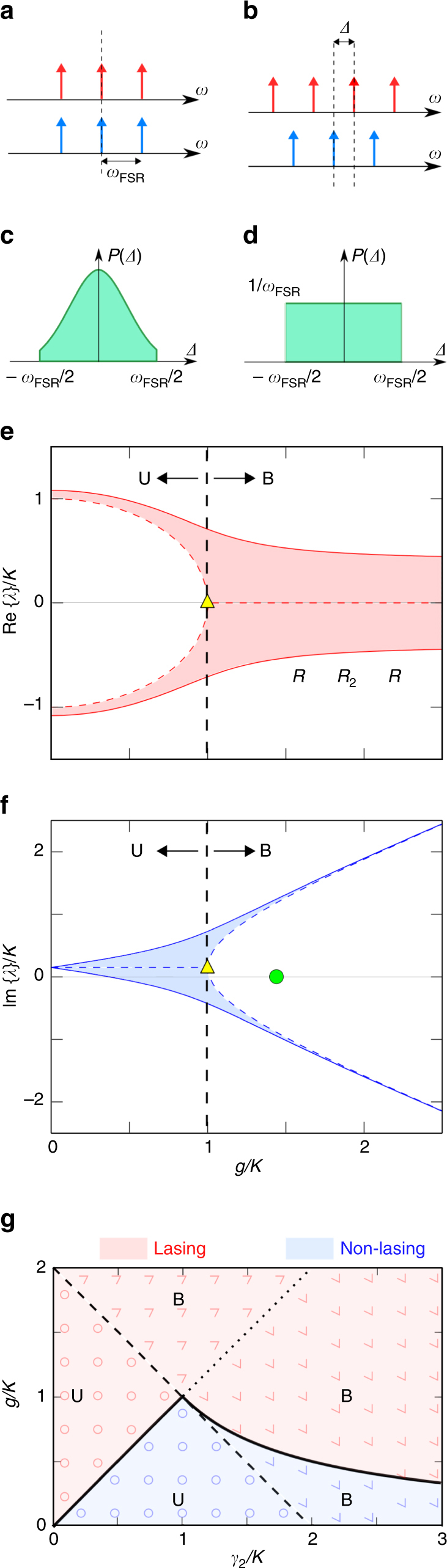
Resonance detuning and its effect on eigenvalue bifurcation. **a**, In the absence of detuning, the resonance frequencies of the gain (red) and lossy (blue) sub-cavities are aligned. **b**, In the presence of detuning Δ ($- {\omega _{{\rm{FSR}}}}/2 < {\rm{\Delta }} < {\omega _{{\rm{FSR}}}}/2$), the sub-cavity resonances are no longer aligned. **c**, **d**, Candidates for the probability distribution *P*(Δ) of the detuning: (**c**) a Gaussian or (**d**) a uniform distribution. **e**, **f**, Trajectories of **e** the real and **f** imaginary components of the eigenvalues *λ*_1,2_ for a linear PT-symmetric configuration *g*+*γ*_1_=*γ*_2_. Dashed curves correspond to Δ=0, whereas the solid curves are for the case ${\rm{\Delta }} = {\omega _{{\rm{FSR}}}}/10$. The shaded regions correspond to all the intermediate detuning values. As the gain *g* increases, the real parts of the eigenvalues Re{*λ*} tend to coalesce whereas the corresponding imaginary parts Im{*λ*} bifurcate. The exceptional point at zero-detuning (yellow triangle) occurs at *g*=*κ*, whereupon Re{λ}=0 and Im{*λ*}=*γ*_1_, thus separating the unbroken (U) and broken (B) PT-symmetric phases. The green circle corresponds to the experimental value for the lasing threshold at *R*_2_=6.8% (Table 1), plotted on the axis to represent gain-clamping. Inset in **e** shows the PT-cavity configuration. **g**, The domains of operation of a structured cavity as dictated by the values of gain *g* and loss *γ*_2_ (*γ*_2_=0). The dotted line *g*=*γ*_2_ corresponds to the PT-symmetric condition. The dashed line *g*+*γ*_2_=2*κ* separates the unbroken (U, *g*<2*κ*−*γ*_2_, represented by circles) and broken (B, *g*>2*κ*−*γ*_2_, represented by edges) domains, according to Eq. (5). The lasing (red) and non-lasing (blue) regions are delineated by a solid line (the lasing threshold). In U, lasing occurs when *g*>*γ*_2_, whereas lasing occurs in B when *g*>*κ* ^2^/*γ*_2_

Here we have introduced an effective temporal coupling coefficient κ between the sub-cavities (to be defined below); *γ*_1_ and *γ*_2_ are temporal linear losses in the amplifying and attenuating sub-cavities, respectively (Supplementary Eqs 18 and 19), which incorporate leakage from the side mirrors and the loss imposed by the VOA; and *g* is the small-signal gain. Here, Δ is the frequency difference between the resonances of the sub-cavities (Fig. 3a, b); henceforth referred to as the detuning. These parameters are all related to the mirror reflectivities and fibre lengths (Supplementary Note 2). We introduce gain saturation in Eq. 2 to capture the power dynamics after the onset of lasing^43^. A useful feature of this model is that it can apply to a wide range of non-Hermitian photonic systems beyond ours.

We are still missing a model for the temporal coupling coefficient κ between the two sub-cavities. To this end, we have adapted the Lamb coupled-cavity model^46, 47^. Lamb modelled the coupling between two coupled cavities through a permittivity bump of infinitesimal thickness, which we relate here to the reflectivity *R*_2_. In addition, leakage from the finite-reflectivity side mirrors effectively produces a shift in the FSR, leading to a dependence of κ on *R*. These considerations lead to an expression for κ,

4 $$\kappa = \frac{{{v_{\rm{g}}}}}{{2n_{\rm{o}}^2d}}\left( {1 - R} \right)\sqrt {\frac{{1 - {R_2}}}{{{R_2}}}} ,$$

where *d* is the fibre-cavity length, *v*_g_ is the group velocity and *n*_o_ is the refractive index (see Supplementary Note 3 for details).

In light of the macroscopic nature of the fibre-based cavity, we assume that the detuning Δ is a random variable. Indeed, given the long cavity length, and thus the extremely small FSR, slight perturbations in the experimental conditions may cause Δ to potentially vary across the whole FSR. The solutions are obtained numerically by carrying out an ensemble average over a distribution for Δ, either a Gaussian distribution $P\left( \Delta \right) \propto \exp \left\{ { - {\Delta ^2}/\left( {2{\sigma ^2}} \right)} \right\}$ (Fig. 3c) or a uniform distribution (Fig. 3d) as candidate models. Analysis under these considerations leads to the intriguing conclusion that features associated with the presence of an exceptional point (a non-Hermitian degeneracy)^48–55^ can–in principle–still be detected.

### Linear model for predicting the lasing threshold

The lasing modes can be obtained from Eqs 2 and 3 in the steady-state. This model is valid both before and after lasing occurs, and to ensure the consistency of our analysis we compare computed lasing thresholds of the PT-symmetric arrangement to those obtained from the linear transfer matrix method (Eq. 1). To achieve this, we linearise Eq. 2 by ignoring gain saturation and set the detuning to Δ=0, and then determine the lasing thresholds by assuming a harmonic ansatz for Eqs 2 and 3 of the form $$\left( {\begin{array}{*{20}{c}}{a\left( t \right)} \\ {b\left( t \right)} \\ \end{array}} \right) = \left( {\begin{array}{*{20}{c}} {{a_0}} \\ {{b_0}} \\ \end{array}} \right){e^{i\lambda t}},$$ where $$\left( {\begin{array}{*{20}{c}} {{a_0}} \\ {{b_0}} \\ \end{array}} \right)$$ is a constant vector. The general solution for the eigenvalues has the form

5 $${\lambda _{1,2}} = - \frac{i}{2}\left( {g - {\gamma _1} - {\gamma _2}} \right) \pm \kappa \sqrt {1 - {{\left( {\frac{{g - {\gamma _1} + {\gamma _2}}}{{2\kappa }}} \right)}^{\!\!\!2}}} .$$

Within the linear, zero-detuning, PT-symmetric configuration (*g*+*γ*_1_=*γ*_2_), the two eigenvalues are ${\lambda _{1,2}} = i{\gamma _1} \pm \sqrt {{\kappa ^2} - {g^2}}$. The lasing threshold is identified by determining the onset for a negative imaginary component of the eigenvalues, ${g_{{\rm{th}}}} = \sqrt {\gamma _1^2 + {\kappa ^2}}$. Computing the lasing threshold based on this model reveals excellent agreement with the predictions of the transfer matrix method for the PT-symmetric structure (Supplementary Fig 3).

The behaviour of the eigenvalues while varying *g* displays a bifurcation, as illustrated in Fig. 3e, f (dashed curves). When *g*<*κ*, the eigenvalues have the same imaginary part *iγ*_1_ but distinct real parts $\pm \sqrt {{\kappa ^2} - {g^2}}$. As *g*→*κ*, the real parts coalesce at zero (Fig. 3e) whereas the imaginary components diverge along forked trajectories (Fig. 3f). We denote the range *g*<*κ* as the unbroken PT-symmetry regime (U), and the range *g*>*κ* the broken PT-symmetry regime (B), separated by the exceptional point at *g*=*κ*. The behaviour of the field is quite distinct in these two regimes (see Supplementary Note 4 for a detailed analysis). The unbroken-PT regime features equal field amplitudes in the two sub-cavities $$\left( {\begin{array}{*{20}{c}}{{a_0}} \\ {{b_0}} \\ \end{array}} \right) = \left( {\begin{array}{*{20}{c}} 1 \\ { \pm {e^{ \pm i\theta }}} \\ \end{array}} \right)$$, where sin *θ*=*g*/*κ*. The power emitted from the gain and loss sub-cavity ports are thus expected to be equal. In the broken-PT regime, the modal field is more concentrated in the gain or loss sub-cavity having unequal amplitudes $$\left( {\begin{array}{*{20}{c}} {{a_0}} \\ {{b_0}} \\ \end{array}} \right) = \left( {\begin{array}{*{20}{c}} 1 \\ {i{e^{ \pm \theta }}} \\ \end{array}} \right)$$, where cosh *θ*=*g*/*κ*, leading to unequal power emission from the two ports. Four sharply delineated domains of operation can be identified while varying the loss and gain independently: lasing in B, lasing in U, non-lasing in B, and non-lasing in U, as depicted in Fig. 3g.

We now consider the impact of detuning Δ on the system while retaining the linear PT-symmetric condition ($G{\cal L} = 1$). As Δ increases, the bifurcation in the real and imaginary parts of the eigenvalues is smeared out in a complementary fashion. Before the EP, the real part closely resembles the zero-detuning results, but deviates considerably after the EP. The opposite is observed in the imaginary part: it closely follows the zero-detuning results after the EP and diverges beforehand. It can be shown on theoretical grounds that the presence of detuning precludes the observation of a pure unbroken-PT mode (Supplementary Note 5). We can nevertheless define a pseudo-unbroken symmetry regime, whereupon $\left| {{a_0}} \right| \approx \left| {{b_0}} \right|$ and the amplitudes are affected in a similar manner upon changing the gain and loss^43^. Note that in a strict PT-symmetric configuration (the dashed zero-detuning curves in Fig. 3e, f), lasing will only occur in the broken-symmetry regime, which has been the case in previous experiments^32, 33^. Nevertheless, the calculations in Fig. 3e, f show that the smearing of the bifurcation resulting from detuning can produce lasing in the unbroken-PT regime (see Supplementary Note 6 for a detailed analysis). Furthermore, this restriction can be relaxed by relying on unbalanced gain and loss ($G{\cal L} \ne 1$; Fig. 3g)^42^.

### Nonlinear steady-state coupled-cavity model for PT-lasing dynamics

Various models have recently been put forth to study the interplay of nonlinearity and PT-symmetry^56–59^. In the structure under consideration, the lasing field dynamics, such as power-scaling with gain, can be analysed using the nonlinear model in Eqs 2 and 3. An important property of lasing structures in the steady-state is that the saturated gain always clamps to the net amount of attenuation present in the system^60^. A critical consequence of this general physical restriction is that the gain/loss contrast no longer determines the transition between different symmetry phases, only the loss does. We confirm this prediction by again employing a harmonic ansatz (with Δ=0) but without imposing a balance between gain and loss. Instead, we regard them as independent variables. By allowing for only real eigenvalue solutions (as a result of gain clamping), we obtain analytical expressions for two distinct phases of field oscillation, which we map to the unbroken and broken PT-symmetry regimes described above:

6 $${\gamma _2} \le \kappa :{\left( {\begin{array}{*{20}{c}}a \\ b \\ \end{array}} \right)_{\!\!\!\rm{U}}} = \sqrt {\frac{g}{{{\gamma _1} + {\gamma _2}}} - 1} \left( {\begin{array}{*{20}{c}}1 \\ { \pm {e^{ \pm i\theta }}} \\ \end{array}} \right){e^{ \pm i\left( {\kappa \cos \theta } \right)t}},$$

7 $${\gamma _2}  >\kappa :{\left( {\begin{array}{*{20}{c}} a \\ b \\ \end{array}} \right)_{\!\!\rm{B}}} = \sqrt {\frac{g}{{{\gamma _1} + {\kappa ^2}/{\gamma _2}}} - 1} \left( {\begin{array}{*{20}{c}} 1 \\ {i\kappa /{\gamma _2}} \\ \end{array}} \right),$$

where the parameter *θ* in Eq. 6 is obtained from sin *θ*=*γ*_2_/*κ*. Two new features emerge here. In contrast to the linear model in which the gain/loss contrast determines the boundary between the broken and unbroken regimes, this boundary in the nonlinear regime is dictated by the loss *γ*_2_ in the lossy sub-cavity alone. The unbroken PT-phase (Eq. 6) is characterised by equal intensities ${\left| a \right|^2} = {\left| b \right|^2}$ in the sub-cavities and the two nonlinear supermodes are split in frequency by 2*κ* cos *θ*. On the other hand, the broken PT-phase entails an unequal distribution of intensities with ${\left| a \right|^2}  >{\left| b \right|^2}$. Another important feature is that the supermodes now exhibit fixed amplitudes because of nonlinearity, dictated by the cavity gain and loss values, in contradistinction to the linear regime. An intuitive explanation of loss-induced enhancement of the lasing power is that the field profile becomes more asymmetric across the system as the loss increases because the lasing mode becomes increasingly localised within the gain side, thereby leading to a rise in the lasing power *I*_Gain_. Eq. (7) showing a broken nonlinear supermode, quantitatively captures this behaviour since the ratio between the steady-state fields in the gain and loss cavities, i.e., *γ*_2_/*κ*, increases as the loss *γ*_2_ increases.

**Fig. 4:**
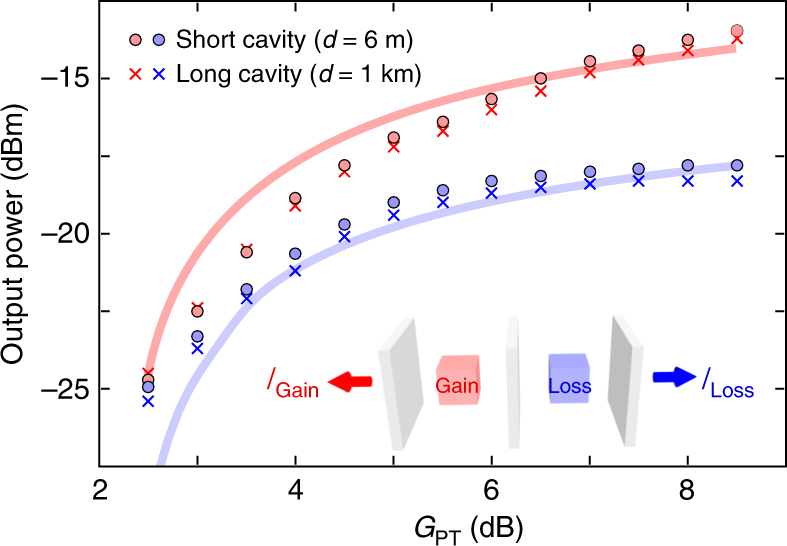
Output power-scaling from a PT-symmetric laser with gain-loss contrast. Plot of the output power from the loss and gain laser cavity ports as the gain-loss contrast is increased while maintaining the PT-symmetric balance $G{\cal L} = 1$; inset shows the cavity configuration. Measured values are shown as circles and crosses for cavity configurations of total lengths *d*=6 m and 1 km, respectively. The solid blue and red curves are simulations of the output power from loss and gain ports, respectively, obtained from the nonlinear model of the coupled fibre system in Eqs 2–3 after making use of measured values for the model parameters and fitting the detuning Δ

In our experiment conducted on a macroscopic fibre system extending for many metres, a pertinent question is whether the observation of such prominent broken and unbroken phases is still possible in the presence of the unavoidable resonance detunings. As a first demonstration of the validity of the nonlinear analysis described above, we measure the power-scaling characteristics of the PT-symmetric laser while holding *R*_2_=6.8% fixed and increasing the gain-loss contrast while maintaining the balance $G{\cal L} = 1$. A unique feature of our experimental arrangement is that the output power from the loss and gain sub-cavity ports (${I_{{\rm{Gain}}}} = {\left| a \right|^2}$ and ${I_{{\rm{Loss}}}} = {\left| b \right|^2}$) can be recorded separately and quantitatively (Fig. 4). It is thus possible to determine unambiguously whether lasing is initiated in the broken or unbroken symmetry phases. We carried out these measurements in two cavity configurations that we denote ‘short’ and ‘long’. In the short cavity, the total length is *d* ≈ 6 m, which is associated with a FSR of *λ*_FSR_=*λ* ^2^/2*nd*=133 fm. The cavity has a quality factor of *Q* = 5.2×10^7^ and a finesse of ${\cal F} = 14$. In the long cavity, we inserted an extra 1-km-long fibre spool in the loss sub-cavity (Fig. 1d), which exacerbates the detuning between the two sub-cavities. The total length is *d* ≈ 1 km, the FSR is *λ*_FSR_=0.8 fm, *Q*=8.7×10^9^ and ${\cal F} = 14$. The data reveals clearly that lasing occurs in the broken regime ${I_{{\rm{Gain}}}} \ne {I_{{\rm{Loss}}}}$ in both cavity configurations. Note however that *I*_Gain_ ≈ *I*_Loss_ at low gain/loss contrasts, which indicates that an unbroken phase is approached, as can be expected from Fig. 3g.

To compare the data on power scaling with predicted values based on the nonlinear model, we must include the impact of phase detuning in the system of Eqs 2 and 3. We compute an ensemble average over a Gaussian distribution for Δ over one FSR; Fig. 3c. The standard deviation *σ* plays an important role in determining the lasing characteristics. We fitted the results of the coupled model for different values of σ and obtained a good match for $\sigma = {\omega _{{\rm{FSR}}}}/10$. This quantifies the amount of average resonance detuning between the two coupled-fibre sub-cavities. Using a uniform probability distribution (Fig. 3d) predicts a substantially larger contrast between *I*_Gain_ and *I*_Loss_ than that observed experimentally.

The trends in Fig. 4 clearly show that the disparity between *I*_Gain_ and *I*_Loss_ continues to grow with *g*, thus confirming that the mode in the gain sub-cavity further localises as the gain-loss contrast in the PT-system is enhanced^5, 15, 32–35^. This is a well-known feature of the broken-PT phase. Since the steady-state always remains in this phase for the balanced values of *γ*_2_=*g*+*γ*_1_ maintained here, we deduce from the results in Eq. 2 that the range over which the loss is varied is actually higher than the coupling strength between the fibre cavities for *R*_2_=7%.

### Observing statistical PT-symmetry breaking

**Fig. 5:**
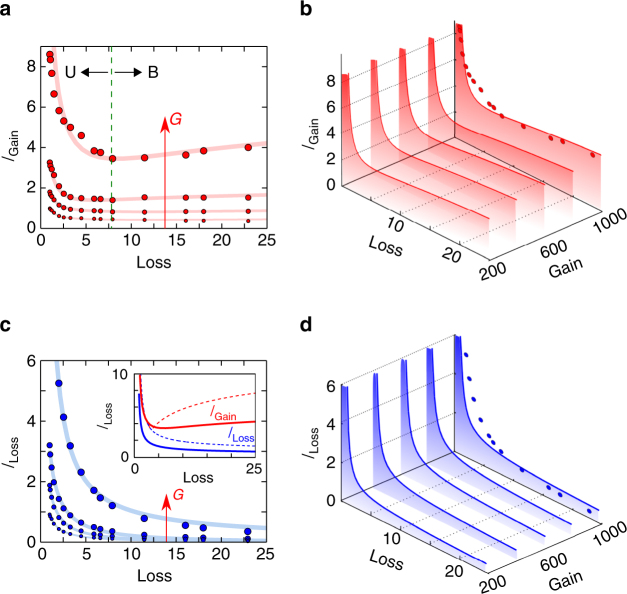
Lasing characteristics of a statistical PT-symmetric cavity around the exceptional point. **a**, Measured values of the output power from the gain port *I*_Gain_ (red circles) while varying the attenuation ${\cal L}$ at different gain values *G* (30, 25, 20 and 15 dB). The solid curves are best fits to guide the eye, with fittings obtained from Eqs 2–3 by a suitable choice of coupling *κ* and detuning Δ. As the loss is gradually increased at fixed gain, *I*_Gain_ is non-monotonic. First *I*_Gain_ decreases with ${\cal L}$ and goes through a minimum at the exceptional point (indicated by the vertical dashed line), and then increases with loss as the gain and loss sub-cavities decouple. **b**, Simulations for *I*_Gain_ while varying the attenuation ${\cal L}$ at different values of *G* using Eqs 2–3, based on experimental parameters. The red circles correspond to the data in the top-most graph in **a** for *G*=30 dB. The area under the curves are shaded to highlight the gradual increase of the lasing power upon increasing gain. **c**, Same as **a** for the power from the loss port *I*_Loss_. Inset shows theoretical plots of *I*_Gain_ and *I*_Loss_ at *G*=30 dB for the statistical PT-symmetric configuration of our experiment (solid curves) and the ideal deterministic configuration (dashed curve, Δ=0) highlighting the bifurcation in output power as a consequence of PT-symmetry breaking upon passing through the exceptional point of the system. **d**, Same as **b** for *I*_Loss_ instead of *I*_Gain_

Finally, we demonstrate that our macroscopic fibre-based laser-cavity system–despite the extreme random detuning between the sub-cavities–still displays the signature of an exceptional point. It is clear from Fig. 3g that transitioning between the unbroken- and broken-symmetry phases associated with a lasing system in the steady state can take place by varying the loss *γ*_2_ alone at fixed gain *g*. Crucially, a quantitative observation of this transition necessitates independent tuning of the gain and loss and unambiguous measurements of the power at the two output ports. Both of these requirements are satisfied in our experimental arrangement. We vary *γ*_2_ via the VOA after holding *g* as provided by the SOA fixed at a value well above the lasing threshold of the gain sub-cavity, such that lasing occurs regardless of *γ*_2_. We have carried out this experiment for four values of single-pass amplification (*G*=15,20,25,30 dB), and for each value we sweep the VOA single-pass attenuation from 0 to 25 dB while recording the lasing power at the two output ports. Increasing the loss results in a monotonic drop in power from the loss port as might be expected (Fig. 5c, d). However, the result for the gain port is counter-intuitive: the power initially drops with increasing loss, but then increases as further loss is added (Fig. 5a, b). This increase in lasing power with additional loss is particularly visible when the gain is held at 30 dB. At a gain of 15 dB, this effect has vanished and a transition is no longer detectable.

Loss-induced enhancement of lasing power has been observed in microcavities and is attributed to the notion of an exceptional point. While the same effect is observed in this macroscopic cavity, it is worth mentioning that strictly pure broken and unbroken phases do not exist in this cavity because the propagation phases are not deterministic. Yet, even in this statistical environment, we have confirmed that a PT-phase transition is still observable in such large-scale active cavities.

We note that a perfect eigenstate coalescence disappears in our system as a consequence of the statistical fluctuations. But this does not have a detrimental effect on the expected response of the system: the behaviour of eigenvalues is smoothed out when the loss *γ*_2_ is varied. In other words, instead of coalescing, the eigenvalues still approach each other around the original location of the EP (associated with the zero-detuning case) and also tend to bifurcate afterwards. This is the reason why a loss-induced enhancement of lasing power is still observable in our randomly varying system (see Supplementary Fig. 5 for details).

## Discussion

Early studies of PT-symmetry focused solely on deterministic models, owing to the micro-scale nature of the coupled resonators and waveguides investigated. In such settings, the impact of a fixed deterministic detuning can be easily understood. In contrast to this scenario, the random phase fluctuations in macroscopic-scale photonic systems lead to drastic detuning between coupled cavities that may span, in principle, the full FSR. Carrying out an ensemble average over a range of outcomes becomes necessary, and it is not clear a priori that the essential features of PT-symmetry will survive.

It is worth mentioning that a complete elimination of detuning between coupled resonators is obviously not possible due to unavoidable fabrication imperfections. However, micro-scale settings afford the benefit of not only restricting the detuning to small values in comparison with the cavity FSR but also of having the detuning approximately invariant over time. We deliberately dispense with such near-ideal configurations in order to study non-Hermitian effects in a statistical system. In the coupled-fibre arrangement considered here, the detuning between the cavity resonances is of the order of the FSR and also fluctuates randomly over time.

In this work, we have presented the realisation of a statistical PT-symmetric lasing structure using coupled fibre cavities extending over a kilometre in length. An important outcome of our experiment has been the persistence of many essential features of the deterministic formulation of PT-symmetry. For example, the lasing threshold was found to be robust against random phase fluctuations. Indeed, the lasing threshold in a PT-symmetric cavity containing a gain-balancing loss can be lower than that of the same cavity after removing the loss and the coupling mirror, thus potentially providing significant benefits compared to a gain-only cavity. In all cases, the experimental results are in agreement with theoretical predictions after ensemble averaging over a Gaussian distribution of detuning values.

With regards to lasing power emerging from the gain and loss ports of our structure, we have observed a transition between the two well-known phases of unbroken and broken symmetry in this statistical system occurring around an exceptional point. The presence of random phase fluctuations; however, prevents a complete coalescence of eigenvalues and thus precludes the observation of a pure unbroken phase where the lasing powers from the gain and loss ports are equal. Crucially, in this nonlinear system the transition behaviour between the unbroken- and broken-symmetry phases is dictated only by the loss in the lossy sub-cavity. Despite the statistical nature of the experimental arrangement, the optical power decays from both the loss and gain cavities with increasing cavity loss until the exceptional point is reached, after which the power counterintuitively begins to rise at the gain port with further increase in the incorporated loss. Such loss-induced transparency and lasing effects have so far been observable only in micro-scale devices. Our results thus indicate that the notion of PT-symmetry–and non-Hermitian optics in general–may have impact on large-scale non-deterministic platforms such as fibre networks.

## Methods

### Experimental arrangement

In the fibre-based cavity, the mirrors are custom-made FBGs on single-mode fibres (SMF28) in the C-band (O-Eland Inc., central wavelength  ≈ 1552.5 nm, bandwidth  ≈ 5 nm). The gain of the SOA is fine-tuned (resolution  < 0.05 dB). Similar fine-tuning for optical attenuation is achieved by cascading the VOA (Thorlabs VOA50PM-APC) with a secondary SOA (Thorlabs BOA1004P), which enables high-resolution adjustment of the net loss in the lossy sub-cavity. The gain spectra of the SOAs were calibrated over the bandwidth of operation (5 nm) using a tunable laser (Agilent 8164A Mainframe with 81680A Tunable Laser Source Module). The VOA employed in our setup has a flat spectrum since it induces loss by physically blocking the optical beam. All fibre pigtails are APC-type to minimise unwanted reflections at the fibre connections. All the SOAs operate in the TE-polarisation mode, and we ensure that the polarisation of the fibre mode is TE when it reaches the SOAs. In all the experimental configurations (except the long cavity), the fibres are polarisation-maintaining and the beam polarisation remains TE when circulating throughout the cavity. For the long cavity, we use polarisation controllers to guarantee that TE-polarisation reaches the SOAs. The output power from each port is recorded by an optical spectrum analyser (OSA, Advantest Q8381A) with a spectral resolution of 0.1 nm.

### Ensemble averaging in the presence of random detuning

The simulation results provided in Figs 4 and 5 for the nonlinear system of Eqs 2–3 were carried out by first finding steady-state values of ${\left| a \right|^2}$ and ${\left| b \right|^2}$ for a specific value of the detuning Δ. We then compute an ensemble average for these steady-state intensities over a full FSR assuming either a Gaussian or a uniform probability distribution for Δ. This is explained in Supplementary Note 7, and the equation involved is stated here for convenience:

8 $$\langle I_{{\rm{a}},{\rm{b}}}^{({\rm{ss}})}\rangle = \!\!\!\mathop {\int}\nolimits_{\!\!\!\!\! - {\omega _{{\rm{FSR}}}}/2}^{{\omega _{{\rm{FSR}}}}/2} {I_{{\rm{a}},{\rm{b}}}^{\left( {{\rm{ss}}} \right)}\left( {\rm{\Delta }} \right)P\left( {\rm{\Delta }} \right)d{\rm{\Delta }}}$$

Here *P*(Δ) is the probability distribution followed by the detuning Δ and $I_{{\rm{a}},{\rm{b}}}^{\left( {{\rm{ss}}} \right)}$ is the steady-state intensity found for a specific value of Δ. To obtain the final curves given in Figs 4 and 5, this procedure is followed for each parameter value over the whole range of the loss *γ*_2_ and gain *g*.

### Data availability

The data that support the plots within this paper and other findings of this study are available from the corresponding author upon reasonable request.

## Electronic supplementary material

Supplementary Information
