# Supplementary material for: Statistical parity-time-symmetric lasing in an optical fibre network
Source: Nat Commun. 2017 Nov 7;8:1359. doi: 10.1038/s41467-017-00958-x (PMC5676724; doi:10.1038/s41467-017-00958-x)
Supplement: Supplementary file 1 — Supplementary Information [file 41467_2017_958_MOESM1_ESM.pdf]

Supplementary Note 1. Calculation of lasing thresholds using transfer matrix method

In this Section, we provide a derivation – based on the transfer matrix method – of the lasing thresholds for the structured cavity configurations compared in the main text (Fig. 2 and Table 1).

Transfer matrix of the cavity

We obtain the lasing threshold by extracting the poles of the transfer matrix of the full optical system. This approach assumes the poles of the transfer matrix cross the real axis of the complex frequency plane when transitioning from sub-lasing to lasing [1,2]. This procedure amounts to finding the conditions at which the transmission coefficient reaches a singularity and thus diverges.

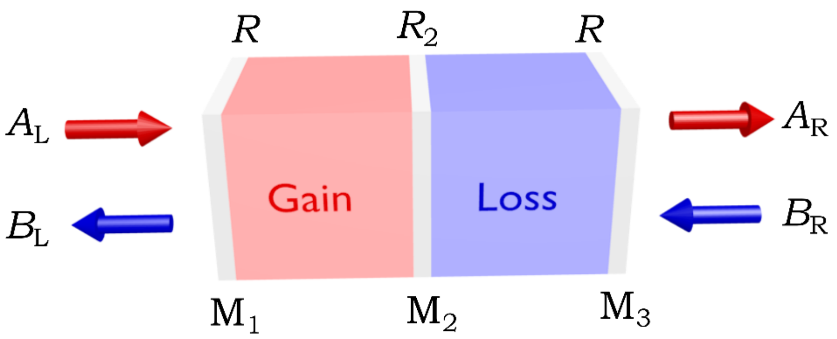

Supplementary Figure 1 | Structured lasing cavity configuration. We depict a schematic of the structured cavity as a two-port system. The mirror M_1, M_2 and M_3 have the reflectivities R, R_2 and R, respectively. The incoming amplitudes from the left and the right are A_L and B_R, respectively. The outgoing amplitudes to the left and the right are B_L and A_R, respectively.

Consider a structured optical cavity comprising two coupled subcavities that contain gain and loss sections, as shown schematically in Supplementary Figure 1. The mirrors used are assumed lossless but are not necessarily symmetric, so that they may be represented in general by a scattering matrix of the form [3]

$$S = e^{i\beta}\begin{pmatrix} t & -re^{i(\beta-\alpha)} \\ re^{-i(\beta-\alpha)} & t \end{pmatrix}. \qquad (1)$$

Here t and r are the (real) transmission and reflection coefficients, respectively, $t^2 + r^2 = 1$, β and α are the transmission and reflection phases for incidence from the left, respectively, and β and $2\beta - \alpha$ are the transmission and reflection phases for incidence from the right, respectively.

The scattering matrices act on incoming fields $\begin{pmatrix} A_{\mathrm{L}} \\ B_{\mathrm{R}} \end{pmatrix}$ to produce the outgoing fields $\begin{pmatrix} A_{\mathrm{R}} \\ B_{\mathrm{L}} \end{pmatrix}$. The reflection and transmission coefficients for the optical power are $R = r^2$ and $T = t^2$, respectively. To satisfy the PT-symmetry condition, M_1 and M_3 must have mirror-symmetry with respect to the centre of the structure. If we take M_1 to be represented by the S-matrix in Eq. S1.1, then this symmetry requirement dictates that the S-matrix for M_3 has the form:

$$S = e^{i\beta}\begin{pmatrix} t & re^{-i(\beta-\alpha)} \\ -re^{i(\beta-\alpha)} & t \end{pmatrix}. \qquad (2)$$

Furthermore, the mirror M_2 must be symmetric, which adds the constraint to its S-matrix that $\beta = \alpha - \pi/2$, such that its S-matrix has the simple form:

$$S = e^{i\beta}\begin{pmatrix} t & ir \\ ir & t \end{pmatrix}. \qquad (3)$$

Exploiting the above-described scattering matrices, we obtain the corresponding transfer matrices that act on the forward and backward fields $\begin{pmatrix} A_{\mathrm{R}} \\ B_{\mathrm{R}} \end{pmatrix}$ on the right to produce the corresponding fields $\begin{pmatrix} A_{\mathrm{L}} \\ B_{\mathrm{L}} \end{pmatrix}$ on the left. The transfer matrices M_1, M_2, and M_3 representing the mirrors are given by

$$M_1 = \frac{1}{t}\begin{pmatrix} e^{-i\beta} & re^{i(\beta-\alpha)} \\ re^{-i(\beta-\alpha)} & e^{i\beta} \end{pmatrix}, \qquad (4)$$

$$M_2 = \frac{1}{t_2}\begin{pmatrix} e^{-i\beta_2} & -ir_2 \\ ir_2 & e^{i\beta_2} \end{pmatrix}, \qquad (5)$$

$$M_3 = \frac{1}{t}\begin{pmatrix} e^{-i\beta} & -re^{-i(\beta-\alpha)} \\ -re^{i(\beta-\alpha)} & e^{i\beta} \end{pmatrix}. \qquad (6)$$

The parameters in these transfer matrices are not subject to statistical fluctuations, except perhaps the phases if the mirrors are implemented by extended fibre Bragg gratings (FBGs).

The transfer matrices for the intervening gain and loss layers are given by

$$M_{\mathrm{G}} = \begin{pmatrix} \frac{1}{\sqrt{G}}e^{-i\varphi_{\mathrm{g}}} & 0 \\ 0 & \sqrt{G}e^{i\varphi_{\mathrm{g}}} \end{pmatrix}, \qquad (7)$$

$$M_{\mathcal{L}} = \begin{pmatrix} \frac{1}{\sqrt{\mathcal{L}}}e^{-i\varphi_{\ell}} & 0 \\ 0 & \sqrt{\mathcal{L}}e^{i\varphi_{\ell}} \end{pmatrix}, \qquad (8)$$

where G and $\mathcal{L}$ are single-pass amplification and attenuation factors for the power traversing the gain and loss layers, respectively, and φ_{g} and φ_{ℓ} are the single-pass propagation phases in the gain

or loss layers, respectively. In the exact PT-symmetric configuration, $G\mathcal{L}=1$ and $\varphi_{\mathrm{g}}=\varphi_{\ell}$. However, statistical fluctuations in a macroscopic cavity precludes the realization of the latter condition.

The total transfer matrix for the whole cavity is thus

$$M = M_1\, M_{\mathrm{G}}\, M_2\, M_{\mathcal{L}}\, M_3 = \begin{pmatrix} m_{11} & m_{12} \\ m_{21} & m_{22} \end{pmatrix}. \tag{9}$$

The cavity transmission, which shares the same poles as the field transmission coefficient, is $T_{\mathrm{L}} = 1/|m_{11}|^2 = 1/M_{11}$, where

$$M_{11} = \frac{1}{(1-R)^2(1-R_2)}\left\{\frac{1}{G\mathcal{L}} + \frac{G}{\mathcal{L}}RR_2 + \frac{\mathcal{L}}{G}RR_2 + G\mathcal{L}R^2 + 2\sqrt{RR_2}\left(\frac{1}{\mathcal{L}} + \mathcal{L}R\right)\cos\varphi_{\mathrm{G}} + 2\sqrt{RR_2}\left(\frac{1}{G} + GR\right)\cos\varphi_{\mathcal{L}} + 2R[\cos(\varphi_{\mathrm{G}}+\varphi_{\mathcal{L}}) + R_2\cos(\varphi_{\mathrm{G}}-\varphi_{\mathcal{L}})]\right\}. \tag{10}$$

Therefore the cavity transmission is determined by the mirror reflectivities R and R_2 and the gain G and the loss $\mathcal{L}$, and (3) two phases φ_{G} and $\varphi_{\mathcal{L}}$:

$$\varphi_{\mathrm{G}} = 2\varphi_{\mathrm{g}} + 2\beta - \alpha + \beta_2 + \pi/2, \tag{11}$$

$$\varphi_{\mathcal{L}} = 2\varphi_{\ell} + 2\beta - \alpha + \beta_2 + \pi/2. \tag{12}$$

Lasing thresholds

Since the cavity length L_{cavity} is very large with respect to the optical wavelength λ, $L_{\mathrm{cavity}} \gg \lambda$, the single-pass phases may not be deterministic due to minute thermal or mechanical fluctuations in the optical fibres used. We thus assume that the cavity single-pass phases are random each with a uniform probability distribution in the interval from 0 to 2π. Consequently, lasing may occur at any wavelength within the gain and loss bandwidth. To obtain the lasing threshold, we hold fixed the physical parameters related to the optical loss and mirror reflectivities, while varying the gain. After obtaining the lasing gain threshold, we can find the special case for the PT-symmetric configuration by choosing the attenuation to satisfy $G\mathcal{L}=1$.

At any value of gain, lasing is initiated whenever the cavity phases produce a zero in M_{11} (a transmission pole). We therefore minimize M_{11} with respect to the phases, and then extract the zeros to find the gain corresponding to the lasing threshold. We thus first set $\frac{\partial M_{11}}{\partial\varphi_{\mathrm{G}}} = \frac{\partial M_{11}}{\partial\varphi_{\mathcal{L}}} = 0$, from which we find a minimal value of M_{11} when $\varphi_{\mathrm{G}}=0$ and $\varphi_{\mathcal{L}}=\pi$,

$$M_{11}|_{\min} = M_{11}(\varphi_{\mathrm{G}}=0,\, \varphi_{\mathcal{L}}=\pi) = \frac{1}{T^2T_2}\frac{1}{G\mathcal{L}}\left[G\left(\mathcal{L}R+\sqrt{RR_2}\right) - \left(1+\mathcal{L}\sqrt{RR_2}\right)\right]^2. \tag{13}$$

From this minimal value of M_{11}, we obtain the zero at a threshold gain G_{th} given by

$$G_{\text{th}} = \frac{1}{\sqrt{R}} \frac{1+\mathcal{L}\sqrt{RR_2}}{\mathcal{L}\sqrt{R}+\sqrt{R_2}}. \tag{14}$$

This general expression for the gain threshold is the minimum required gain for lasing to occur, while all other parameters are fixed except for the phases that are assumed to vary randomly.

We can now obtain expressions for the gain threshold when special structures are of interest. First, for a pseudo-PT-symmetric structure with $G\mathcal{L} = 1$, the following threshold holds for lasing:

$$G_{\text{PT}} = \frac{1-R}{2\sqrt{RR_2}} + \sqrt{1 + \left(\frac{1-R}{2\sqrt{RR_2}}\right)^2}, \tag{15}$$

which was used in the main text (Eq. 1). Second, when we eliminate the attenuation in the loss subcavity $\mathcal{L} = 1$, the gain threshold G_0 has the form

$$G_0 = \frac{1}{\sqrt{R}} \frac{1+\sqrt{RR_2}}{\sqrt{R}+\sqrt{R_2}}. \tag{16}$$

Finally, when the lossy subcavity is altogether open, corresponding to $\mathcal{L} = 0$, the gain threshold G_{open} has the usual form

$$G_{\text{open}} = \frac{1}{\sqrt{RR_2}}. \tag{17}$$

Supplementary Note 2. Non-Hermitian temporal coupled-mode equations

We now introduce the temporal coupled-mode equations used as a basis for the analysis presented in the main text. We consider an optical-fibre-based laser cavity, comprising two coupled sub-cavities (one including net gain and the other net loss) of length L each and group velocity v_g. We introduce time-dependent quantities to characterize the sub-cavities: a linear background loss per second γ_1 for the field in the gain sub-cavity (which only depends on the side mirror M_1 of reflectivity R) and a corresponding quantity γ_2 for the loss sub-cavity (which depends on the side mirror M_3 of reflectivity R in addition to the imposed attenuation). Furthermore, we define a temporal small-signal gain g for the gain sub-cavity, which is dictated by the SOA amplification. These temporal losses and the small-signal gain are given by

$$\gamma_1 = \frac{v_g}{2L} \ln\left(\frac{1}{\sqrt{R}}\right), \tag{18}$$

$$\gamma_2 = \frac{v_g}{2L} \left[\ln\left(\frac{1}{\Gamma\sqrt{R}}\right)\right], \tag{19}$$

$$g = \frac{v_g}{2L} \ln(G), \tag{20}$$

where G and Γ are the single-pass intensity amplification and attenuation factors that are set by the SOA and the VOA, respectively.

Based on these considerations, we define two temporal coupled-mode equations for the mean-field amplitudes $\tilde{a}$ and $\tilde{b}$ in the amplifying and attenuating fibre sub-cavities, respectively, which adequately capture all the essential features of the optical field dynamics in the coupled fibre-cavity system:

$$\frac{d\tilde{a}}{dt} = -\gamma_1\tilde{a} + i\frac{\Delta}{2}\tilde{a} + \left(\frac{g}{1+|\tilde{a}|^2/I_s}\right)\tilde{a} + i\kappa\tilde{b}, \tag{21}$$

$$\frac{d\tilde{b}}{dt} = -\gamma_2\tilde{b} - i\frac{\Delta}{2}\tilde{b} + i\kappa\tilde{a}. \tag{22}$$

We have introduced into this model the nonlinear gain-saturation, the loss mechanisms, and the coupling between the sub-cavities, where I_s is the gain-saturation intensity and Δ is used to phenomenologically introduce detuning between the resonances of the sub-cavities. For simplicity, we normalize the field amplitudes with respect to the saturation value by introducing the scaled amplitudes $(\tilde{a}, \tilde{b}) = \sqrt{I_s}(a, b)$, whereupon we obtain the coupled-mode equations:

$$\frac{da}{dt} = -\gamma_1 a + i\frac{\Delta}{2}a + \left(\frac{g}{1+|a|^2}\right)a + i\kappa b, \tag{23}$$

$$\frac{db}{dt} = -\gamma_2 b - i\frac{\Delta}{2}b + i\kappa a. \tag{24}$$

Supplementary Note 3. Extended Lamb model for coupled cavities

In traditional configurations of coupled cavities, such as evanescently coupled micro-ring resonators [4], one considers time-averaged energy amplitudes in each cavity. The temporal coupling between the cavities is dependent on the effective interaction region between them. In our setup for coupled fibre cavities, their interaction is mediated by the middle mirror M_2, which is a lumped element (Fig. 1 and Supplementary Fig. 2). The usual approach of extracting a distributed effective coupling is thus no longer valid. A more pertinent method was employed by W. E. Lamb [5,6] in treating coupled-laser configurations, in which the central mirror coupling two sub-cavities (Supplementary Fig. 2a) was modelled as a bump of width w in the permittivity distribution in space along the cavity axis z (Fig. S2b). The permittivity of the so-called bump is defined as:

$$\varepsilon(z) = \varepsilon_0\left({n_0}^2 + \rho\, \delta(z)\right), \tag{25}$$

where n_0 is the refractive index of the background medium. Lamb's well-known model thus connects the sought-after coupling coefficient κ (Eqs. 2-3 in the main text and Supplementary Note 2 and Supplementary Notes 4-6 below) to the bump parameter ρ:

$$\kappa = \frac{v_g}{k\rho L}, \tag{26}$$

according to Eq. 18 of reference [4]; here L is the length of a single sub-cavity, $k = n_0\, 2\pi/\lambda$, and v_g is the group velocity of light in the fibres.

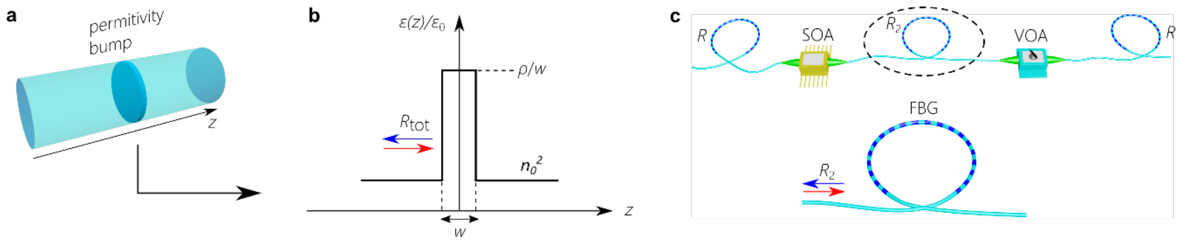

Supplementary Figure 2 | Extended Lamb model to extract the coupling coefficient. a, Cavity used in the Lamb model in which a discontinuity (a permittivity "bump") is introduced in a laser cavity. **b**, Model for the permittivity bump of width w. The bump permittivity is represented by ρ as of the Supplementary Equation (25). **c**, The PT-symmetric laser configuration used in our experiments. The middle FBG M_2 corresponds to the permittivity bump in Lamb's model.

In our experiment, the bump represents the mirror M_2 of reflectivity R_2. By establishing a relationship between the bump parameter ρ and the reflectivity R_2, we can then express κ in terms of R_2. This connection enables us to simulate the dynamics of our structure via coupled-mode theory after employing the proper coupling coefficient.

According to the Supplementary Fig. 2b, the discontinuity in the refractive index ($n = \sqrt{\rho/w}$) results in Fresnel reflection at each interface, R_{int}:

$$R_{\text{int}} = \left(\frac{n-n_0}{n+n_0}\right)^2 = \left(\frac{\sqrt{\rho/w}-n_0}{\sqrt{\rho/w}+n_0}\right)^2 \cong 1 - 4n_0\sqrt{w/\rho}, \tag{27}$$

Here the subscript ‘int’ indicates interface and the approximation makes use of the fact that $n = \sqrt{\rho/w}$ grows in the limit $w \to 0$. The two interfaces of the bump define a Fabry-Pérot resonator with a reflection of R_{tot} (Supplementary Fig. 2b):

$$R_{\text{tot}} = 1 - \frac{1}{1+\left[\frac{2\sqrt{R_{\text{int}}}}{1-R_{\text{int}}}\sin\left(\frac{2\pi}{\lambda}nw\right)\right]^2}, \tag{28}$$

Taking the limit $w \to 0$ leads to the approximation $\sin(\pi n w/\lambda) = \sin(\pi\sqrt{\rho w}/\lambda) \cong \pi\sqrt{\rho w}/\lambda$, and substituting R_{int} from Supplementary Equation 21 into Supplementary Equation 24, so that the total reflection of the bump is:

$$R_{\text{tot}} \cong \frac{1}{1+\left(\frac{n_0\lambda}{\pi\rho}\right)^2}. \tag{29}$$

We associate the reflectivity of the mirror R_2 with that of the bump-based model R_{tot}, $R_{\text{tot}} \to R_2$. Therefore ρ can now be expressed in terms of R_2,

$$\rho = \frac{n_0\lambda}{\pi}\sqrt{\frac{R_2}{1-R_2}}. \tag{30}$$

According to Lamb’s model the coupling rate is related to ρ (and thus to R_2) via

$$\kappa = \frac{v_g}{k\rho L} = \frac{v_g}{2n_0^2 L}\sqrt{\frac{1-R_2}{R_2}}, \tag{31}$$

Coupled-mode theory simulations that make use of κ defined in this last expression do not quite agree with our measurements. The reason is that the Lamb model assumes that the out-coupling windows M_1 and M_3 are perfect, which is not the case in our configuration. In another seminal paper, W. E. Lamb considered the case of partially transmitting windows that radiate into free space [6]. The analysis revealed that for windows with a high enough reflectivity (as is the case in our experiment), only a small leakage and a change in the cavity free-spectral-range (FSR) are effectively introduced – whereas the modal profiles are left unchanged. The change in the FSR

means that the effective length of the cavity is now dependent upon the out-coupling mirror reflectivity. We model this effect in the expression for the coupling between the two sub-cavities of the PT-laser as follows:

$$\kappa = \frac{v_g}{2n_0^2 L}(1-R)\sqrt{\frac{1-R_2}{R_2}}. \tag{32}$$

This is the definition of κ used in our coupled-mode theory simulations throughout. The value of κ obtained as such, when used to find the lasing threshold g_{th} based on Equations (2-3) of the main text, agrees well with the threshold found based on a transfer matrix analysis as provided in section Supplementary Note 1. When one considers the scenario of $G\Gamma = 1$, γ_2 is always greater than g, in fact $\gamma_2 = \gamma_1 + g$ – see Supplementary Equations 19 and 20. Now the condition for lasing in an unbroken mode is given by, $g > (\gamma_1 + \gamma_2)$ which can never be achieved (consider the imaginary part of Supplementary Equation 59). However, the lasing threshold in the broken mode might be surpassed. In this case when one enters the broken symmetry regime where the square root in Supplementary Equation 59 turns complex, the threshold can be obtained from the requirement: $\gamma_1 - \kappa\sqrt{\left(\frac{g}{\kappa}\right)^2 - 1} < 0$. This then leads to,

$$g_{\text{th}} = \sqrt{\gamma_1^2 + \kappa^2}. \tag{33}$$

Translating this into a single-pass gain value using Eq. S2.3, one obtains,

$$G_{\text{th}} = \exp\left\{\sqrt{\left[\ln\left(\frac{1}{\sqrt{R}}\right)\right]^2 + \left[\frac{(1-R)}{n_0^2}\sqrt{\frac{1-R_2}{R_2}}\right]^2}\right\}. \tag{34}$$

The value of this threshold is compared to G_{PT} (according to Supplementary Equation 11) for various values of R_2 in the figure below. A good agreement between the two is apparent.

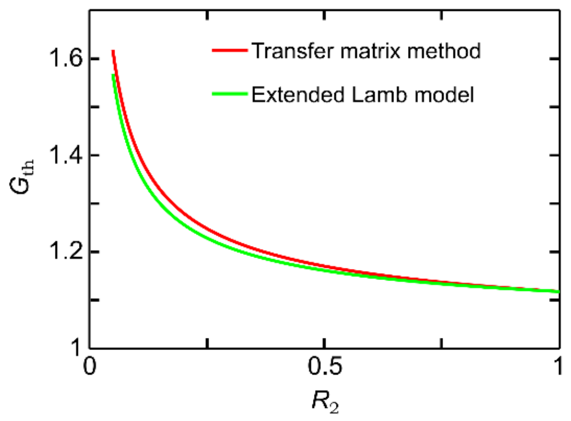

Figure S3 | Comparison of the lasing thresholds for the two presented models. PT-symmetric lasing lasing thresholds are obtained from the transfer matrix method and the extended Lamb model**,** and are plotted as a function of R_2 with both out-coupling mirror reflectivities assumed to be $R = 80\%$.

Supplementary Note 4. Temporal coupled-mode solutions in absence of detuning

We first obtain solutions to the coupled-mode equations (Supplementary Equations 23-24) in absence of detuning, $\Delta = 0$ to obtain the coupled-mode equations:

$$\frac{da}{dt} = -\gamma_1 a + \left(\frac{g}{1+|a|^2}\right) a + i\kappa b, \qquad (35)$$

$$\frac{db}{dt} = -\gamma_2 b + i\kappa a. \qquad (36)$$

We seek a harmonic solution of the form $\begin{pmatrix} a(t) \\ b(t) \end{pmatrix} = \begin{pmatrix} a_0 \\ b_0 \end{pmatrix} e^{i\lambda t}$, where λ is real and $\begin{pmatrix} a_0 \\ b_0 \end{pmatrix}$ is a constant vector. When $|a_0| = |b_0|$, we have a so-called unbroken PT-symmetric phase, otherwise, when $|a_0| \neq |b_0|$ we obtain a broken phase. By substituting this solution into Supplementary Equations 35-36 we obtain

$$i\lambda + \gamma_1 - g_s = i\kappa \frac{b_0}{a_0}, \qquad (37)$$

$$i\lambda + \gamma_2 = i\kappa \frac{a_0}{b_0}, \qquad (38)$$

where, as before, $g_s = \frac{g}{1+|a_0|^2}$ for the saturated gain, and we assume that $|a_0|, |b_0| \neq 0$. Multiplying Supplementary Equations 37 and Supplementary Equations 37, we obtain a quadratic equation in λ,

$$\lambda^2 - i(\gamma_1 + \gamma_2 - g_\text{s})\lambda - \kappa^2 - \gamma_2(\gamma_1 - g_\text{s}) = 0. \qquad (39)$$

This quadratic equation has a real solution for λ only when $\gamma_1 + \gamma_2 = g_\text{s}$, whereupon

$$|a_0|^2 = \frac{g}{\gamma_1+\gamma_2} - 1, \qquad (40)$$

and the solutions for λ take the simple form:

$$\lambda^2 = \kappa^2 - \gamma_2^2 \rightarrow \lambda_{1,2} = \pm\kappa\sqrt{1-\left(\frac{\gamma_2}{\kappa}\right)^2}, \qquad (41)$$

which applies when $\gamma_2 \leq \kappa$. By defining $\gamma_2 = \kappa \sin\theta$, we obtain a concise expression for the eigenvalues: $\lambda_{1,2} = \pm\kappa\cos\theta$.

To obtain the complex ratio between b_0 and a_0, we define $b_0 = a_0 \alpha e^{i\phi}$ and subtract Supplementary Equation 38 from Supplementary Equation 37,

$$-2\gamma_2 = i\kappa\left(\alpha e^{i\phi} - \frac{1}{\alpha e^{i\phi}}\right), \qquad (42)$$

which leads to

$$2i\sin\theta = \alpha\cos\phi + i\alpha\sin\phi - \frac{1}{\alpha}\cos\phi + i\frac{1}{\alpha}\sin\phi. \qquad (43)$$

Comparing the real parts of Supplementary Equation 43 yields

$$\alpha \cos\phi = \frac{1}{\alpha}\cos\phi \;\rightarrow\; \alpha = \pm 1. \tag{44}$$

Now, comparing the imaginary parts of Eq. Supplementary Equation 43 we have

$$2\sin\theta = \alpha\sin\phi + \frac{1}{\alpha}\sin\phi. \tag{45}$$

We now have a pair of conditions $\alpha = 1 \rightarrow \phi = \theta$ and $\alpha = -1 \rightarrow \phi = -\theta$. Hence, the steady-state solution is given by

$$\binom{a}{b} = \sqrt{\frac{g}{\gamma_1+\gamma_2} - 1}\,\binom{1}{\pm e^{\pm i\theta}}\, e^{\pm i(\kappa\cos\theta)t}, \tag{46}$$

where θ is obtained from $\sin\,\theta = \gamma_2/\kappa$. We therefore obtain a pure unbroken PT-symmetric phase when there is no detuning, that is valid when $\gamma_2 \le \kappa$ and $g > \gamma_1 + \gamma_2$. The latter restriction is inferred from Supplementary Equation 40. On the other hand, if $\gamma_2 > \kappa$, we have stationary steady-state solutions $\lambda = 0$ in Supplementary Equation 37-38,

$$0 = -\gamma_1 + \frac{g}{1+|a_0|^2} + i\kappa\frac{b_0}{a_0}, \tag{47}$$

$$0 = -\gamma_2 + i\kappa\frac{a_0}{b_0}. \tag{48}$$

Supplementary Equation 48 implies that $b_0 = i\frac{\kappa}{\gamma_2}a_0$, which we substitute in Supplementary Equation 47 to obtain

$$|a_0|^2 = \frac{g}{\gamma_1+\kappa^2/\gamma_2} - 1. \tag{49}$$

The solution within this regime is thus given by,

$$\binom{a}{b} = \sqrt{\frac{g}{\gamma_1+\kappa^2/\gamma_2} - 1}\,\binom{1}{i\kappa/\gamma_2}, \tag{50}$$

which corresponds to a PT-symmetry-broken phase solution that is valid when $\gamma_2 > \kappa$ and $g > \gamma_1 + \kappa^2/\gamma_2$. The last condition is a consequence of having a positive absolute value $|a_0|^2$ in Supplementary Equation 49.

In summary, steady-state solutions of the coupled-mode equations Supplementary Equation 35-36 have been found in two different regimes:

(I) Unbroken: when $g > \gamma_1 + \gamma_2$ and $\ge \gamma_2 \rightarrow \binom{a}{b} = \sqrt{\frac{g}{\gamma_1+\gamma_2} - 1}\,\binom{1}{\pm e^{\pm i\theta}}\, e^{\pm i(\kappa\cos\theta)t}$;

(II) Broken: when $g > \gamma_1 + \kappa^2/\gamma_2$ and $< \gamma_2 \rightarrow \binom{a}{b} = \sqrt{\frac{g}{\gamma_1+\kappa^2/\gamma_2} - 1}\,\binom{1}{i\kappa/\gamma_2}$.

Supplementary Note 5. Proof for the absence of a pure unbroken-symmetry mode in presence of detuning

In the presence of detuning $\Delta \neq 0$, it can be shown that a formal unbroken PT-symmetric phase (that is, one with equal intensities of harmonic fields at the two output ports $|a_0| = |b_0|$) cannot be realized. This fact can be deduced from Supplementary Equation 23-24 where a harmonic solution $\begin{pmatrix} a \\ b \end{pmatrix} = \begin{pmatrix} a_0 \\ b_0 \end{pmatrix} e^{i\lambda t}$ with $\lambda \in \mathbf{R}$ results in the following equations:

$$\lambda = i\gamma_1 + \frac{\Delta}{2} - ig_s + \kappa \frac{b_0}{a_0}, \tag{51}$$

$$\lambda = i\gamma_2 - \frac{\Delta}{2} + \kappa \frac{a_0}{b_0}, \tag{52}$$

where $g_s = g/(1 + |a_0|^2)$. From Supplementary Equation 52, we obtain the ratio between a_0 and b_0,

$$b_0 = i \frac{\kappa}{\gamma_2 + i\delta} a_0, \tag{53}$$

where $\delta = \lambda + \frac{\Delta}{2}$. Substituting in Supplementary Equation 51, and assuming that $a_0 \neq 0$, we obtain

$$\left\{\gamma_1 - g_s + \frac{\kappa^2 \gamma_2}{\gamma_2^2 + \delta^2}\right\} + i\left\{\delta - \Delta - \frac{\kappa^2 \delta}{\gamma_2^2 + \delta^2}\right\} = 0. \tag{54}$$

The imaginary part of this equation implies that

$$\frac{\gamma_2^2 + \delta^2}{\kappa^2} = \frac{\delta}{\delta - \Delta}. \tag{55}$$

Achieving the unbroken PT-symmetric phase requires $|b_0| = |a_0|$, which necessitates (via Supplementary Equation 53) that

$$\frac{\kappa^2}{\gamma_2^2 + \delta^2} = 1 \quad \rightarrow \quad \frac{\delta - \Delta}{\delta} = 1 \tag{56}$$

Such a result is only possible in one of two scenarios: (1) $\Delta = 0$, that is, no detuning; or (2) $\delta \rightarrow \infty$. Hence for a non-zero detuning, the exact unbroken PT phase in the two coupled sub-cavities never appears.

Supplementary Note 6. Linear analysis in the presence of detuning

Before the onset of lasing in the coupled-cavity structure, a linear analysis of the field dynamics can be carried out because the intensities are small. Under these conditions, the dynamical equations can be cast in the form:

$$\frac{da}{dt} = -\gamma_1 a + i\frac{\Delta}{2}a + ga + i\kappa b, \quad (57)$$

$$\frac{db}{dt} = -\gamma_2 b - i\frac{\Delta}{2}b + i\kappa a. \quad (58)$$

Detuning leads to an avoided eigenvalue-coalescence, as we proceed to show. First, we consider the harmonic ansatz $\begin{pmatrix} a(t) \\ b(t) \end{pmatrix} = \begin{pmatrix} a_0 \\ b_0 \end{pmatrix} e^{i\lambda t}$ in absence of detuning, i.e. $\Delta = 0$, to obtain:

$$\lambda_{1,2} = -\frac{i}{2}(g - \gamma_1 - \gamma_2) \pm \kappa\sqrt{1 - \left(\frac{g-\gamma_1+\gamma_2}{2\kappa}\right)^2}. \quad (59)$$

whereupon a second order bifurcation takes place in the imaginary parts of the eigenvalue λ at $g = 2\kappa + \gamma_1 - \gamma_2$ by gradually increasing the gain from a small value. The system is said to enter a PT-symmetry broken regime when the amount of gain increases beyond this point. That is, the unbroken PT-symmetry regime occurs when $g < \gamma_1 - \gamma_2 + 2\kappa$ (the eigenvalues have not undergone a bifurcation) and the broken PT-symmetry regime when $g > \gamma_1 - \gamma_2 + 2\kappa$ (the eigenvalues have bifurcated). This transition point between broken and unbroken PT-symmetry regimes is better known as an exceptional point (EP) [7-9]. At this point, the two eigenvalues and associated eigenvectors $(a_0, b_0)^{\mathrm{T}}$ coalesce:

$$\lambda_{1,2} = i(\gamma_2 - \kappa), \quad |1,2\rangle = \begin{pmatrix} 1 \\ i \end{pmatrix}. \quad (60)$$

In general, lasing occurs once the sign of the imaginary part of one of the eigenvalues $\lambda_{1,2}$ becomes negative, $\mathrm{Im}\{\lambda\} < 0$, per our harmonic ansatz. Because the imaginary parts of the eigenvalues drop monotonically with g prior to the EP (Supplementary Equation 59), the expression for the eigenvalues at the EP (Supplementary Equation S60) dictates whether lasing is initiated in the unbroken or broken PT-symmetry regimes. If $\mathrm{Im}\{\lambda\} < 0$ at the EP because $\kappa > \gamma_2$, this indicates that lasing has already started before the EP was reached. In other words, lasing initiates here in the unbroken PT-symmetry regime. On the other hand if $\mathrm{Im}\{\lambda\} > 0$ at the EP because $\kappa < \gamma_2$, then lasing has not started when the EP is reached. Therefore, lasing initiates after the EP in the broken PT-symmetry regime. These two scenarios of lasing occurring in the unbroken and broken PT-symmetric phases are depicted in Supplementary Fig. 4.

This picture of pure unbroken- and broken-symmetry regimes is true only for a zero-detuned system ($\Delta = 0$). Once detuning is introduced ($\Delta \neq 0$), the eigenvalues do not coalesce, as shown in Supplementary Fig. 5. In other words, an exact EP does not occur. Nevertheless, two regimes are still identifiable, one in which the eigenvalues approach each other (unbroken PT-symmetry) and the other where they diverge (broken PT-symmetry). In the presence of detuning $\Delta \neq 0$, the eigenvalues are

$$\lambda_{1,2} = -\frac{i}{2}(g - \gamma_1 - \gamma_2) \pm \kappa\sqrt{1 - \left(\frac{g-\gamma_1+\gamma_2+i\Delta}{2\kappa}\right)^2}. \qquad (61)$$

We can infer from the expression under the square root in Supplementary Equation 61 that $\lambda_1 \neq \lambda_2$ when $\Delta \neq 0$ because the parameters g, γ, and κ are all real. Using $g - \gamma_1 + \gamma_2 + i\Delta = 2\kappa \sin\theta$, where θ is now a complex angle, the eigenvectors associated with these eigenvalues are

$$|1\rangle = \begin{pmatrix} 1 \\ e^{i\theta} \end{pmatrix}, |2\rangle = \begin{pmatrix} 1 \\ -e^{-i\theta} \end{pmatrix}. \qquad (62)$$

Note that the EP in the zero-detuning scenario corresponds to $\theta = \pi/2$.

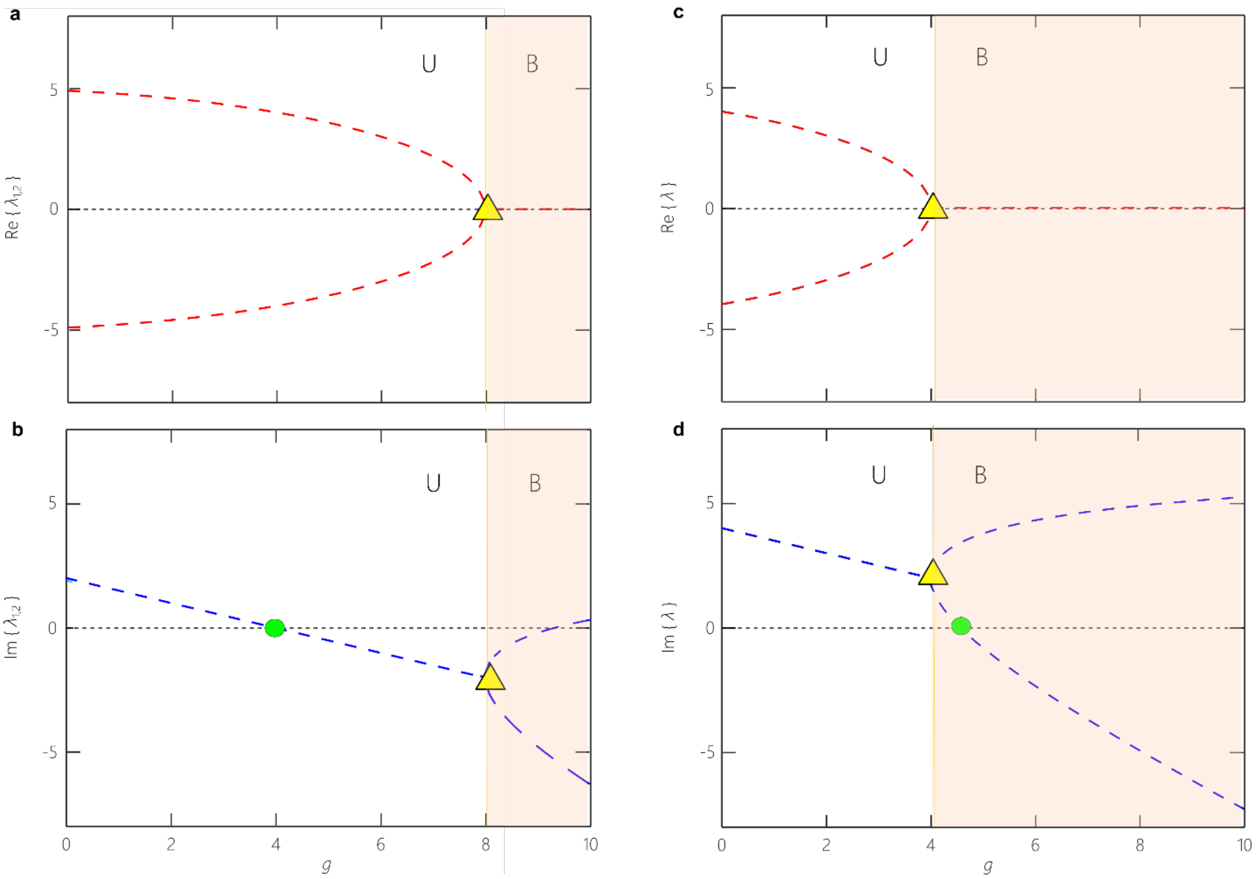

Supplementary Figure 4 | Eigenvalue dynamics, EPs, and lasing in two coupled sub-cavities at zero-detuning. a-d, Trajectories of the two eigenvalues λ_1 and λ_2 of the harmonic solutions to the temporal coupled-mode theory treatment in absence of detuning. The real parts are depicted in the top row and the imaginary parts in the bottom row. The light and dark backgrounds identify the unbroken (U) and broken ('B') PT-symmetric regimes, respectively. EP is depicted as a yellow triangle (where $g = 2\kappa + \gamma_1 - \gamma_2$) and the green circle indicates the point where lasing occurs. In all cases, we use the parameter values $\gamma_1 = 1$ and $\kappa = 5$. **a-b**, When the loss $\gamma_2 = 3$ is less than the coupling strength κ, lasing occurs in the unbroken regime. **c-d**, When the loss $\gamma_2 = 7$ is larger than the coupling strength κ, lasing is initiated in the broken regime instead.

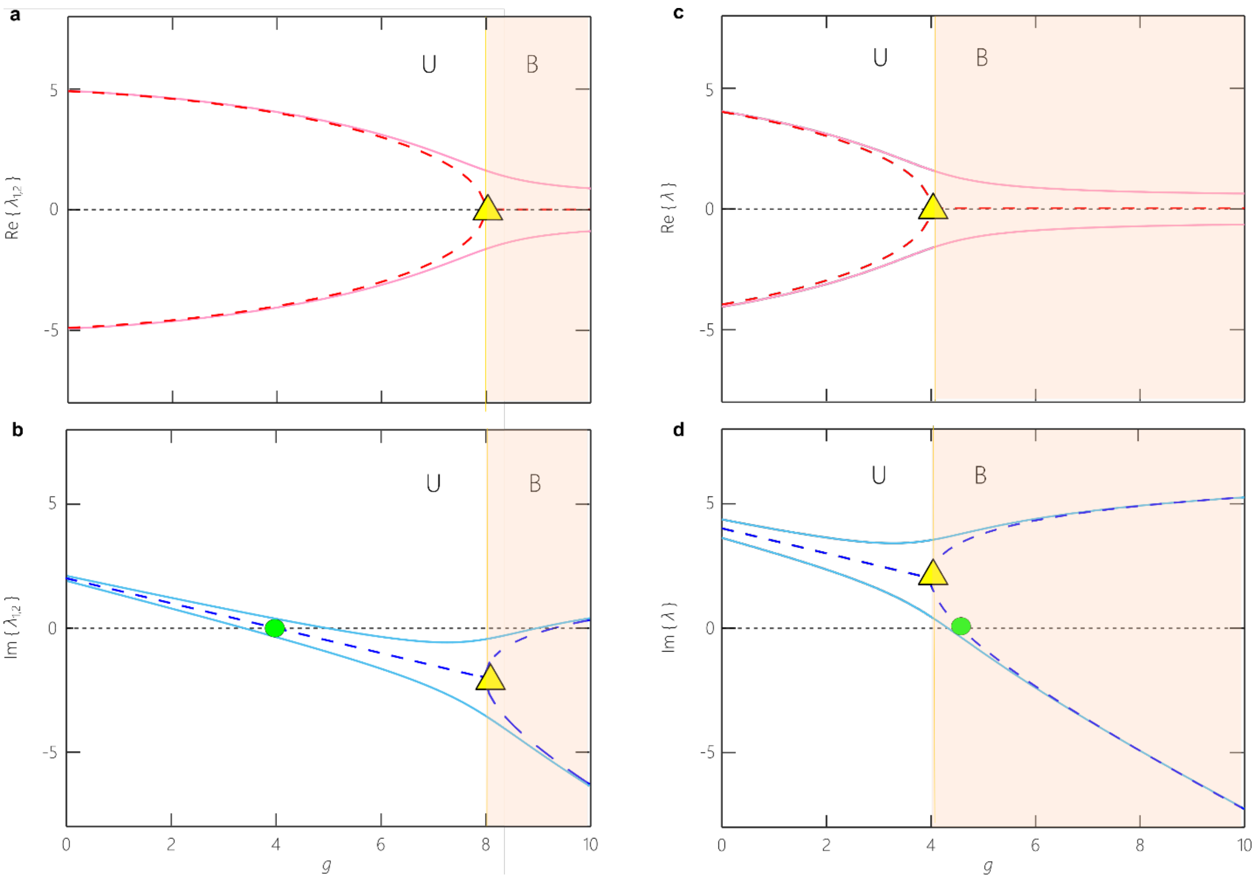

Supplementary Figure 5 | Eigenvalue dynamics, EPs, and lasing in two coupled sub-cavities in presence of detuning. a-d, Trajectories of the two eigenvalues λ_1 and λ_2 of the harmonic solutions to the temporal coupled-mode theory treatment for $\Delta \neq 0$. The real parts are depicted in (a) and (c) and the imaginary parts in (b) and (d). Dashed and solid curves correspond to the eigenvalues in absence and presence of detuning, respectively. The light and dark backgrounds identify the unbroken (U) and broken (B) PT-symmetric regimes, respectively. EP is depicted as a yellow circle and the green circle indicates the point where lasing occurs in the corresponding zero-detuning case In all cases, we use the parameter values $\gamma_1 = 1$, $\kappa = 5$, and $D = 1$. **a-b**, When the loss $\gamma_2 = 3$ is less than the coupling strength κ, lasing occurs in the unbroken regime. **c-d**, When the loss $\gamma_2 = 7$ is larger than the coupling strength κ, lasing is initiated in the broken regime instead.

The real parts of the two eigenvalues are plotted in Supplementary Fig. 4 and Supplementary Fig. 5 against gain g in the case where γ_2 is kept fixed at $\gamma_2 = 3$, the coupling is $\kappa = 5$, and the loss is $\gamma_1 = 1$. The two eigenvalues are initially well apart – but with increasing g they gradually coalesce at the exceptional point (EP) where $g = 2\kappa - \gamma_2 + \gamma_1$. In contrast, the imaginary components of the eigenvalues stay together before the EP and only bifurcate afterwards. If we choose a value of γ_2 higher than κ, $\gamma_2 = 7$, the location of the EP now shifts to the positive imaginary half. In Supplementary Fig. 4c, the initial separation between Re$\{\lambda\}$ is now smaller whereas in Supplementary Fig. 4d, the final distance between Im$\{\lambda\}$ is now much larger compared to the previous case. Indeed, Im$\{\lambda\}$ crossing the zero value and switching sign (the green

circle) heralds the onset of lasing which takes place in the unbroken symmetry mode (U) in Supplementary Fig. 4a-b while in Supplementary Fig. 4c-d it instead occurs in the broken symmetry mode (B).

Interestingly, the transition point between these behaviours is still the same value of gain as the EP, i.e. $g = 2\kappa + \gamma_1 - \gamma_2$, when detuning is introduced (Supplementary Fig. 5). We conclude that, even in the presence of detuning, lasing can be either initiated in an unbroken PT-like mode if the loss in the attenuating component of the coupled cavity configuration is less than the effective coupling, i.e. $\gamma_2 < \kappa$; or in an a broken PT-like mode for the case $\gamma_2 > \kappa$. More importantly, the two PT-symmetric phases have a physical significance in the following sense: Unbroken symmetry implies that the fields in both components of the system behave in a similar fashion, i.e. with increasing gain (loss), $|a|^2$ and $|b|^2$ increase (decrease) in synchrony. However, in the broken symmetry domain, a disparity exists between the behaviours of the fields in the two components. Specifically, the ratio between the field intensity in the amplifying and lossy components $|a|^2/|b|^2$ starts to increase with gain. The bifurcation in the imaginary parts of the eigenvalues after the EP indicates this, as shown in Supplementary Fig. 5.

The scope of the linear analysis presented in this section is not just limited to determining the lasing conditions, but also has important ramifications on the full nonlinear response of the system. The two distinct regimes of unbroken and broken PT-symmetry still manifest themselves in the presence of random frequency detuning and gain saturation. Remarkably, the transition between the two still arises at the point where the loss in the attenuating cavity equals the coupling strength $\gamma_2 = \kappa$. This behaviour is depicted in Fig. 5 of the main text where instead of gain, the loss γ_2 is gradually increased from a low to a very high value including a passage through the point $\gamma_2 = \kappa$. In affirmation of this statement, the intensity emitted from the amplifying sub-cavity displays a dip at this point.

Supplementary Note 7. Probability distribution for detuning

The term that describes the resonance detuning between the coupled subcavities (that is, Δ in Supplementary Equations 23-24) occurs because of random perturbations in the fibres that have their origin in thermal or mechanical fluctuations. Since the fibres are relatively long (> 6 m), length expansions or contractions can easily lead to changes in the path length on the order of a micrometre, which in turn can cause a phase accumulation of 2π. This would result in a frequency detuning equivalent to half the free spectral range (ω_{FSR}) of a single fibre subcavity, as shown in Fig. S6.

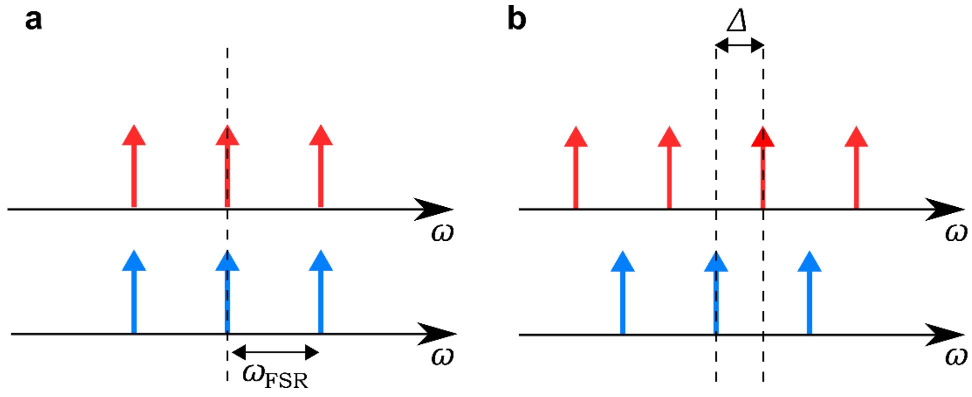

Supplementary Figure 6 | Resonances of the sub-cavities in absence and presence of detuning. a, The resonances of the two sub-cavities are aligned identically in absence of detuning. The cavity free spectral range is ω_{FSR}. **b**, In presence of detuning Δ, the resonance frequencies of the two coupled sub-cavities do not align.

In principle, Δ can span the entire range $-\omega_{\mathrm{FSR}}/2 \leq \Delta \leq \omega_{\mathrm{FSR}}/2$. Two possible probability distributions for Δ are the uniform (Fig. S7a) and Gaussian (Fig. S7b) distributions. Because the detuning-phase-accumulation spans the range $(-\infty, \infty)$ in the Gaussian distribution, we fold the probability distribution function after each period of π equivalent to $\omega_{\mathrm{FSR}}/2$.

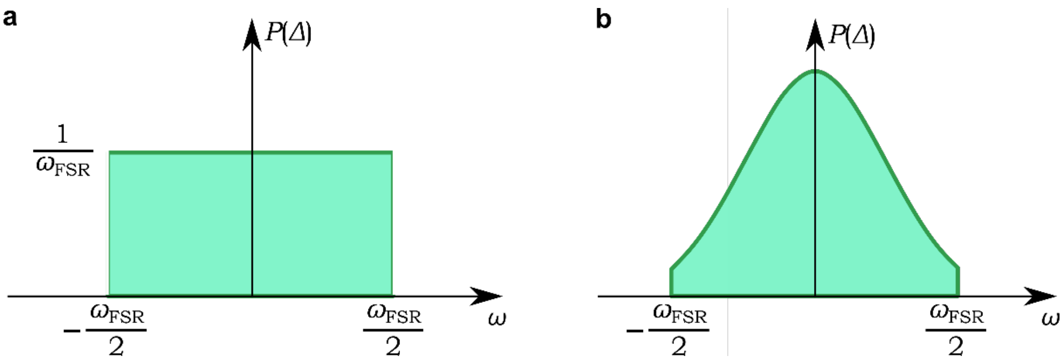

Supplementary Figure 7 | Probability distributions $P(\Delta)$ for the detuning Δ between the two coupled sub-cavities. a, A uniform probability distribution of width ω_{FSR}. **b**, A folded Gaussian probability distribution.

The Gaussian probability distribution function is $P(\Delta) = 1/(\sigma\sqrt{2\pi})\exp(-\Delta^2/2\sigma^2)$, where the full width half maximum (FWHM) is given by $2\sigma\sqrt{2\ln 2}$. To obtain solutions of the system of Equations (2-3) of the main text, we take an ensemble average of the steady state intensities over all the values of Δ, taking into account the relevant probability distribution,

$$\langle I_{\mathrm{a,b}}^{(\mathrm{ss})}\rangle = \int_{-\omega_{\mathrm{FSR}}/2}^{\omega_{\mathrm{FSR}}/2} I_{\mathrm{a,b}}^{(\mathrm{ss})}(\Delta)P(\Delta)d\Delta, \quad (63)$$

In Supplementary Equation 63, $I_{\mathrm{a,b}}^{(\mathrm{ss})}(\Delta)$ is the steady-state (ss) intensity value obtained from a Runge-Kutta simulation for a given set of parameters $(\gamma_1, \gamma_2, g, \kappa, \Delta)$, i.e. while Δ is considered deterministic. It is only after we obtain $I_{\mathrm{a,b}}^{(\mathrm{ss})}(\Delta)$ for all values of Δ, that the averaging is carried out according to Supplementary Equation 63. For computational convenience, this integral can also be approximated by a summation after considering a finite number of sampling points for Δ in the interval $[-\omega_{\mathrm{FSR}}/2, \omega_{\mathrm{FSR}}/2]$. Upon analyzing the numerical results and the experimental data, we found the Gaussian distribution to be a significantly better match when compared with the uniform distribution. Moreover, the standard deviation was also found to be small, i.e. $\sigma \sim 0.1\omega_{\mathrm{FSR}}$. The main text shows results that correspond only to the Gaussian distribution, e.g. Fig. 5. A figure similar to that when one considers a uniform distribution for Δ, is shown in Supplementary Fig. 8. Qualitatively, the uniform distribution leads to a larger split in the gain and loss cavity intensities since the two cavities are in essence decoupled from each other most of the time. On the other hand, a phase transition around the exceptional point more clearly visible in the case of a narrow Gaussian distribution as shown in Fig. 5 of the main text.

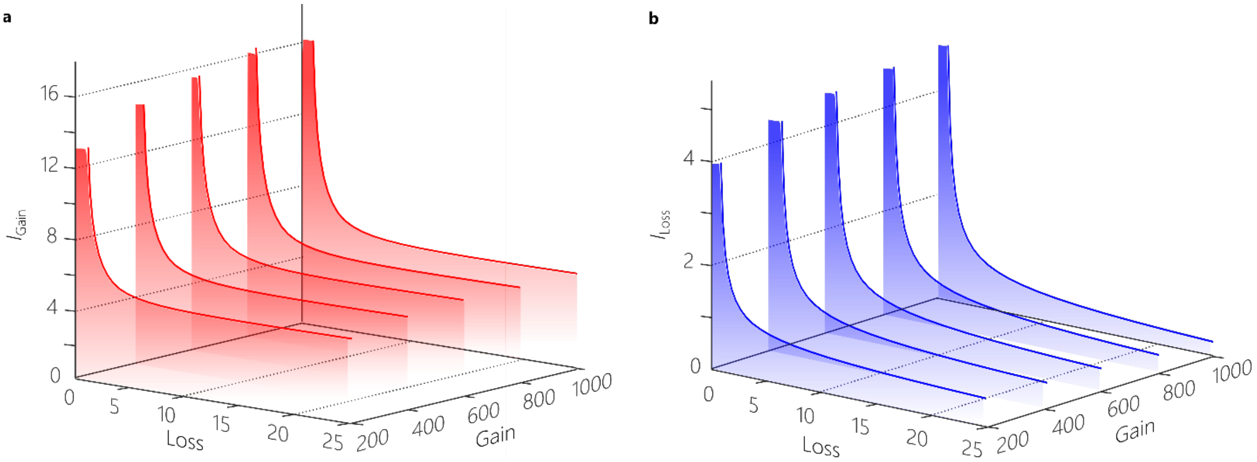

Supplementary Figure 8 | Output lasing powers assuming a uniform distribution for the detuning Δ. a, Simulations for I_{Gain} at different values of G obtained from Equations 2-3 of the main text, based on a uniform probability distribution for Δ. **b**, Same as (**a**) but for power from the loss port I_{Loss}.

Supplementary References

[1] Yeh, P. & Yariv, A. *Photonics: Optical Electronics in Modern Communication* 6th edn., Ch. 6 (Oxford Uni. Press, 2007).

[2] Longhi, S. PT-symmetric laser absorber. *Phys. Rev. A* **82**, 031801(R) (2010).

[3] Jahromi, A. K. & Abouraddy, A. F. Observation of Poynting's vector reversal in an active photonic cavity. Optica **3**, 1194-1200 (2016).

[4] Little, B. E., Chu, S. T., Haus, H. A., Foresi, J. & Laine, J.-P. Microring resonator channel dropping filters. *J. Lightwave Technol.* **15**, 998–1005 (1997).

[5] Spencer, M. B. & Lamb, W. E. Theory of two coupled lasers. *Phys. Rev. A* **5**, 893–898 (1972).

[6] Spencer, M. B. & Lamb, W. E. Laser with a transmitting window. *Phys. Rev. A* **5**, 884–892 (1972).

[7] Kato, T. *Perturbation theory for linear operators* 1st edn. (Springer, Berlin, 1966).

[8] Heiss, W. D. Repulsion of resonance states and exceptional points. *Phys. Rev. E* **61** 929 (2000).

[9] Guo, A. *et al.* Observation of PT-symmetry breaking in complex optical potentials. *Phys. Rev. Lett.* **103**, 093902 (2009).
